# Theoretical guarantees for phylogeny inference from single-cell lineage tracing

**DOI:** 10.1073/pnas.2203352120

**Published:** 2023-03-16

**Authors:** Robert Wang, Richard Zhang, Alex Khodaverdian, Nir Yosef

**Affiliations:** ^a^Algorithms and Complexity Group, David R. Cheriton School of Computer Science, University of Waterloo, Waterloo ON N2L 3G1, Canada; ^b^Department of Electrical Engineering and Computer Sciences, University of California, Berkeley, CA 94720; ^c^Center for Computational Biology, University of California, Berkeley, CA 94720; ^d^Department of Systems Immunology, Weizmann Institute of Science, Rehovot 7610001, Israel

**Keywords:** computational phylogenetics, single-cell lineage tracing, Crispr-Cas9

## Abstract

Lineage-tracing technologies based on Clustered Regularly Interspaced Short Palindromic Repeats and CRISPR-associated protein 9 (CRISPR-Cas9) genome editing have emerged as a powerful tool for investigating development in single-cell contexts, but exact reconstruction of the underlying clonal relationships in experiment is complicated by features of the data. These complications are functions of the experimental parameters in these systems, such as the Cas9 cutting rate, the diversity of indel outcomes, and the rate of missing data. In this paper, we develop two theoretically grounded algorithms for the reconstruction of the underlying single-cell phylogenetic tree as well as asymptotic bounds for the number of recording sites necessary for exact recapitulation of the ground truth phylogeny at high probability. In doing so, we explore the relationship between the problem difficulty and the experimental parameters, with implications for experimental design. Lastly, we provide simulations showing the empirical performance of these algorithms and showing that the trends in the asymptotic bounds hold empirically. Overall, this work provides a theoretical analysis of phylogenetic reconstruction in single-cell CRISPR-Cas9 lineage-tracing technologies.

## Significance

The recent application of CRISPR-Cas9 barcode editing technologies, by allowing for the continuous accumulation of information-rich mutations, has greatly increased the scale and resolution of studies tracking the developmental lineages of single cells. The inference of the unobserved lineage relationships from mutations observed in cells at one terminal time point is a rich computational problem made more difficult by distinct properties of these data that hinder classical phylogenetic approaches. To address these methodological needs for single-cell CRISPR-Cas9 lineage-tracing applications, we provide a theoretical analysis in this domain with the following key contributions: A robust generative model, algorithms with theoretical guarantees on accurate reconstruction, theoretically motivated experimental design principles, and simulations characterizing the empirical conditions for accurate reconstruction.

Recent progress in Lineage-tracing technologies based on Clustered Regularly Interspaced Short Palindromic Repeats and CRISPR-associated protein 9 (CRISPR-Cas9) genome editing now enables the inference of cellular lineage relationships at single-cell resolution in complex organisms where visual observation is not possible. This is owing to CRISPR-Cas9’s ability to generate heritable, irreversible, and information-rich mutation events that can be read-through single-cell assays (such as RNA-seq) and subsequently used to infer the underlying cellular phylogeny (1–10). Typically, these technologies start by engineering a single progenitor cell with artificial transcribed recording sites that accumulate stable insertions or deletions (“indels”) as a result of repair of Cas9 double-stranded breaks. These indel mutations are subsequently inherited by future descendants, and the accumulation of these mutations is used to infer the clonal relationships between the observed cells, stratifying them into clades of increasing resolution. Thus far, studies have paired these technologies with single-cell transcriptomic profiling (11) to study questions in development (e.g., inferring lineage relationships between cellular compartments) (1–5, 7, 10) and cancer progression (e.g., inferring rates and routes of metastases) (8, 9).

An outstanding goal is developing methods to accurately infer the underlying phylogeny and to determine under what experimental conditions the problem is tractable. Exact reconstruction of the ground truth single-cell phylogenetic tree, defined here as having a reconstructed tree that exactly matches that of the ground truth clonal relationships, is complicated by various features of the data. First, convergent evolution (or homoplasy) events can occur whereby the cells might appear to be incorrectly related to each other because the same indel occurs in unrelated clades (6, 12, 13). Second, during the experiments, information at recording sites can be lost due to partial RNA capture, recording site resection, or transcriptional silencing (1, 2, 6, 14, 15). Finally, a mistuned Cas9 editing rate can lead to scenarios where there is a lack of mutation information sufficient for discerning relationships between clades: Either all recording sites being exhausted too early or mutation events being too infrequent (15). As these complications are dependent on experimental parameters, we aim to identify optimal parameters to allow for accurate reconstruction of trees from single-cell lineage-tracing data.

To our knowledge, there has yet to be any work exploring guarantees for exact reconstruction in a general single-cell CRISPR-Cas9 lineage-tracing model. Previous studies have established theoretical guarantees for phylogenetic reconstruction under the Cavender–Farris–Neyman (CFN) 2-state model, in which algorithms have been developed to exactly reconstruct subtrees when there are at least $O(\frac{log(n)}{\ell^{2}})$ characters (16–18), where *n* is the number of taxa and ℓ is the minimum edge length. The CFN model cannot capture the CRISPR-Cas9 settings because there are only two states, mutations are reversible, and there are no missing data (19, 20). Theoretical guarantees exist under perfect phylogeny (6, 21), but this regime is highly improbable in lineage-tracing settings. Other methods like maximum-likelihood (14, 22), parsimony-based (4, 6, 23, 24) and distance-based (25–28) methods rely on heuristics rather than correctness of reconstruction. While there have been results regarding exact reconstruction for neighbor-joining, in particular, given certain error bounds between the true and observed distance metrics (29, 30), there has yet to be work characterizing how and when mutation-based distances meet these criteria. Additionally, while existing studies have used simulations to provide insight into the relationship between reconstruction accuracy and experimental parameters (6, 15, 31), there has been limited theoretical exploration into the single-cell CRISPR-Cas9 lineage-tracing experimental design and how to optimize parameters in order to achieve accurate reconstruction.

In this paper, we perform a theoretical analysis of two algorithms and show that exact reconstruction can be achieved with high probability given a sufficient number of recording sites (characters) in a lineage-tracing experiment. We begin with an algorithm that achieves exact reconstruction with high probability when there are at least $O(\frac{log(n)}{\ell^{2}})$ characters, where ℓ is the minimum edge length, thus matching the guarantees for the CFN 2-state model. The lower-bound assumption translates to a reasonable assumption over the minimal time until cell division (32). We further extend this algorithm and bound to account for missing data, showing that the same bounds still hold assuming a constant probability of missing data. We then give a bottom–up algorithm that achieves high probability of exact reconstruction with $O(\frac{log(n)}{\ell })$ characters, assuming that all edge lengths are between ℓ and $O(\sqrt{\ell })$. The upper bound corresponds to an assumption on the maximum time until cell division, which can be evaluated in lineage-traced populations as they, by definition, should not be postmitotic. We characterize the dependence between the necessary number of characters and other experimental parameters, such as the Cas9 mutation rate controlled by guide affinity (5) and the probability of collision given the indel outcome distribution, thus offering insight into how experimental design may be improved as the field develops. Lastly, via a large set of simulations, we present empirical bounds on the minimum number of characters required for exact reconstruction and show that the theoretical relationships between that number and the other experimental parameters are still present in the empirical bounds.

Our results provide a theoretical analysis of the feasibility of phylogeny inference in the single-cell CRISPR-Cas9-based setting. As the field continues to grow and generate excitement as exemplified by the recent Allen Institute Dream Challenge (31), we anticipate that this work will guide future lineage-tracing studies in both technology development and experimental design.

## Problem Setup and Model Assumptions

In order to tackle the problems of guarantees on exact reconstruction and optimizing experimental design, it is helpful to consider a more abstract theoretical model. We begin with a single cell with *k* unmutated characters, corresponding to *k* unedited recording sites at which CRISPR-Cas9 can induce mutations. This cell then undergoes cell division. Over time, characters randomly mutate from their unedited states at instantaneous rate *λ* according to a Poisson process. This Poisson model of Cas9 mutation reflects recent technologies (5, 6, 8, 9) in which the mutations that are used for lineage tracing occur stochastically and Cas9 and its guides are continuously expressed beginning from the original progenitor cell. We further assume that this mutation rate is constant across all cells in the phylogeny and does not change with time, which may not necessarily be the case due to possible reduction in the expression of Cas9 throughout the course of an experiment.

When a mutation occurs, the respective character adopts a state (corresponding to an indel) according to some probability distribution over the space of possible indels. We assume that once a character mutates, it can never change its state again and that this mutation will be inherited by all descendants of this cell. This irreversibility assumption is derived from the fact that after an indel is introduced at a recording site by Cas9, the guide RNA no longer has affinity at that site, thus preventing future edits (15, 33). After a set period of time has elapsed, a subset of the contemporary cells (leaves of the tree) are collected for sequencing. We denote the size of this set by *n*. Finally, some proportion with expectation *p*_*d*_ of each cell’s characters will have their states rendered indeterminable. These missing data may be due to low capture at the sequencing step and affect only one cell, or through resection (excision) or transcriptional silencing events which are inherited throughout the cell division process and persist in all descendant cells (1, 2, 6, 15, 22). We refer to the former as stochastic missing data and the latter as heritable missing data.

Given the set of samples collected at the end of the experiment, the goal is to construct the phylogenetic tree that relates the observed cells to one another, based on their character/state information. Note that even though we collect a subset of the cells, the underlying “ground truth” phylogeny is still a binary tree. Formally, this problem can therefore be viewed as a character-based inference of a rooted phylogeny where the tree is binary, the root is unmutated, the mutations are irreversible, and some mutation data may be missing. In addition to offering algorithms to solve this problem, our goal is to shed light on the relationship between the various experimental parameters *k*, *q*, *n*, ℓ, *λ*, and *p*_*d*_, finding the regimes in which exact lineage reconstruction is tractable. We next explore the generative model in more detail.

### Generative Process.

Let 𝒯 be a binary tree representing the ground truth phylogeny in a single-cell CRISPR-Cas9 lineage-tracing experiment. Let *S* be the set of leaves in 𝒯, with each leaf representing a single cell in our input. Each edge (*u*, *v*) of 𝒯 has a length, *l*(*u*, *v*), representing the duration of time between the two respective cell division events. Furthermore, we define the distance *dist*(*u*, *v*) between two vertices *u*, *v* as the sum of the lengths of the edges in the path between *u* and *v*. In addition, we denote the root node by *r* and say that a vertex *u* is at depth *d* if *dist*(*r*, *u*) = *d*. Finally, we assume that the distance between the root and any leaf is equal to 1 (normalizing arbitrary time units), as all leaves are sampled at one time point, making the tree ultrametric.

Each node in the tree has *k* independently evolving characters, each of which can take on states in {0, 1, ...*m*}. Each character starts in an unedited state (0) at the root. Once a cell acquires a mutation at a certain character, this mutation is inherited by all descendants of that cell, and further mutations cannot occur at that character in these descendants (irreversibility). For each character, the time it takes for a mutation to occur on a path in the tree is exponentially distributed with rate *λ* and is independent of cell division events. That is, if *r* = 0^*k*^ is the root, then the probability that a mutation occurs along the path from *r* to some downstream descendant vertex *u* for any particular character is $\int_{0}^{dist(r,u)}\lambda e^{-\lambda t}dt=1-e^{-\lambda dist(r,u)}$.

**Fig. 1.**
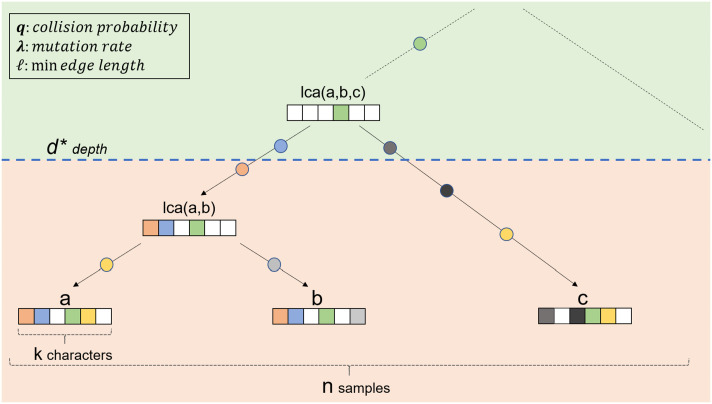
General problem setup. In particular, we note that *a* and *b* are the ingroup, whereas *c* is the outgroup. In addition, we point out some of the key variables used throughout this manuscript: d^*^, *k*, *n*, *q*, *λ*, and ℓ.

We assume that each character mutates independently of all other characters. Additionally, although in practice, target sites can be engineered to have different rates of Cas9 cutting (5), as a simplifying assumption, we take all characters to have the same mutation rate. Once a character mutates, it takes on state *j* ∈ {1, ...*m*} with probability *q*_*j*_. Let $q=\sum_{j=1}^{m}q_{j}^{2}$ be the probability that two independent mutations at the same character index arrive at the same state. At the end of the experiment, each character in the leaves has a *p*_*d*_ probability of becoming indeterminable and adopting the “missing” state. Finally, another measure of similarity between nodes, which we will use throughout, is derived from their mutation profiles. Here, we define by *s*(*u*, *v*) the number of mutations shared by nodes (cells) *u* and *v*. The primary variables used in our analysis are summarized in Fig. 1.

### Additional Definitions and Formulas.

For a triplet of nodes *a*, *b*, *c* ∈ 𝒯, we use the notation of (*a*, *b*|*c*) to denote that *c* is the “outgroup,” namely that *LCA*(*a*, *b*) is a descendent of *LCA*(*a*, *b*, *c*), where *LCA* gives the lowest common ancestor in 𝒯. This leads us naturally to the concept of a triplet oracle.

*Definition 1* (Triplet Oracle): We say that a function *O* : *S*^3^ → *S* is a triplet oracle if for every leaf triplet (*a*, *b*|*c*) ∈ *S*, *O*(*a*, *b*, *c*) = *c*.

It is known that 𝒯 can be reconstructed exactly in *O*(*n* log *n*) time given a triplet oracle (34). Typically, it is unreasonable to expect to have exact triplet oracles in practical applications, so instead, we define a relaxed version of this oracle.

Definition 2 ((ℓ^*^, d^*^)–accurate partial oracle): We say that *O* is an (ℓ^*^, d^*^)−accurate partial oracle, if for every triplet (*a*, *b*|*c*) such that *depth*(*LCA*(*a*, *b*, *c*)) ≤ d^*^, then *O*(*a*, *b*, *c*) returns either *c* or *Null*. In addition, if it also follows that *dist*(*LCA*(*a*, *b*),*LCA*(*a*, *b*, *c*)) ≥ ℓ^*^, then the oracle is guaranteed to return the correct answer, i.e., *O*(*a*, *b*, *c*) = *c*.

In this study, we treat ℓ^*^ ∈ [0, 1] and d^*^ ∈ [0, 1] as error tolerance parameters that can be tuned to the researcher’s liking. The partial oracle does not return the wrong answer for triplets whose LCA is close to the root (max depth d^*^), so if researchers are particularly interested in accurate reconstruction of early events in the phylogeny, they can set d^*^ to the timing of those events. If in addition the LCA and ingroup ancestor of a triplet is separated by a distance of at least ℓ^*^, it is guaranteed to return the correct outgroup. In the remaining cases (e.g., triplets with an LCA far from the root, which are thus more difficult to resolve), the partial oracle can be wrong. Making ℓ^*^ smaller increases the resolution by allowing more poorly separated triplets to be resolved correctly, but as we show in this paper, this comes at the cost of needing more characters. Making d^*^ closer to 1 increases the resolution of the tree in the sense that relationships deeper in the tree will be correct but also requires more characters.

Throughout the first part of this paper, we will simplify the analysis by making the mild assumption that *λ*ℓ^*^ ≤ 1. This is always possible as *λ* can be tuned and ℓ^*^ is a user-defined parameter. We additionally believe that this assumption is not restrictive. For reasonable error tolerance of the reconstructed phylogeny, a researcher would be likely interested in values of ℓ^*^ ≪ 1, and given a small ℓ^*^, a choice of $\lambda >\frac{1}{\ell^{*}}$ is not very practical. While it is the case that a choice of *λ* ≫ 1 would allow exact reconstruction of 𝒯 up to d^*^ with fewer characters when d^*^ is small (shown below), not enough mutations would be left to resolve the lineages beyond d^*^. This is as the exponential model for mutations implies that in expectation, most characters have already mutated by time *Ω*(1/*λ*) in any given lineage. Hence, most choices of *λ*ℓ^*^ will meet this assumption automatically.

Throughout the paper, we will be using the following versions of Hoeffding’s inequality:

If *Y* ∼ *Bin*(*n*, *p*), and *μ* = *np* = *E*(*Y*), then we have:

$$\begin{array}{cc} Pr[Y\geq (1+\beta )\mu ] & \leq exp(-\frac{\beta^{2}\mu }{2+\beta })\;\;\;\;\text{for}\;\beta >0\\ Pr[Y\leq (1-\beta )\mu ] & \leq exp\left(-\frac{\beta^{2}\mu }{2}\right)\;\;\;\;\text{for}\;\beta \in \left(0,1\right). \end{array}$$

## Results

In the first section of this paper, we show that in an experiment where each sample has a sufficiently high number of characters and states (where the required number of characters and states depends on *λ*, *q*, ℓ^*^, d^*^ and *p*_*d*_), it is possible to construct (ℓ^*^, d^*^)−partial oracles with high probability. Given these partial oracles, we have top–down algorithms that can exactly reconstruct 𝒯 up to depth d^*^, either with or without missing data. In particular, note that if d^*^ = 1 and ℓ^*^ ≤ ℓ, where ℓ^*^ is the minimum edge length on 𝒯, then an (ℓ,1)−partial oracle is a triplet oracle which leads to an exact reconstruction of 𝒯 in *O*(*n*log*n*) time. In this paper, we study guarantees in full and partial exact reconstruction of the ground truth tree. We will give the guarantees about full reconstruction as corollaries of our main theorems as follows:

Corollary 1 *Given a ground truth tree, 𝒯, of height normalized to 1 under the lineage-tracing model with *k* characters, *n* samples, constant mutation rate and state space, and minimum edge length ℓ satisfying *λ*ℓ ≤* 1, *there exists a polynomial time algorithm to reconstruct 𝒯 with high probability when* $k=O(\frac{\log n}{\ell^{2}})$.

Accounting for the possibility of missing data (incomplete information on the mutation profile of cells), we get

Corollary 2 *Under the same conditions above, assume that information on the state of any given character in a given cell can be masked with probability *p*_*d*_ independently for each character. In that case, there exist polynomial time algorithms to reconstruct 𝒯 with high probability when* $k=O(\frac{\log n}{\ell^{2}{\left(1-p_{d}\right)}^{3}})$.

As another extension, we consider a less constrained case with no lower bound on edge length. We show that in that case, we can still get partial recovery as follows:

Corollary 3 Under the same conditions as in corollary 1 and given an error tolerance threshold of ℓ^*^ such that *λ*ℓ^*^ ≤ 1, there exists a polynomial time algorithm that with high probability will return a tree which correctly resolves all triplets, (*a*, *b*|*c*) such that *dist*(*LCA*(*a*, *b*, *c*),*LCA*(*a*, *b*) ≥ ℓ^*^ when $k=O(\frac{\log n}{\ell^{*2}})$.

Finally, we consider a more constrained case, where edge lengths are both upper and lower bounded. We demonstrate that it is possible to achieve a stronger lower bound on the number of characters required via a bottom–up algorithm. This part is based on an alternative strategy, in which we conduct a bottom–up tree reconstruction without using an oracle. This alternative approach gives the following theoretical guarantee:

Corollary 4 *If we assume that edge lengths are between ℓ and* $O(\sqrt{\ell })$, *then for a sufficiently low mutation rate, there exists polynomial time algorithms to reconstruct 𝒯 with high probability when* $k=O(\frac{\log n}{\ell })$.

### Partial Reconstruction of Phylogenies with Top–Down Oracle-Based Algorithms.

For a triplet *a*, *b*, *c*, suppose WLOG that *s*(*a*, *b*) ≥ *s*(*b*, *c*) ≥ *s*(*a*, *c*). Our goal is to define a sufficiently high threshold *t* such that if *s*(*a*, *b*) − max(*s*(*b*, *c*),*s*(*a*, *c*)) > *t*, then with high probability, *c* is the outgroup. Since leaf node similarities can be readily computed from our input, this will help define a triplet oracle.

First, consider the case in which there are no missing data and all character states are determinable at the end of the experiment. Let (*a*, *b*|*c*) be a triplet where *dist*(*LCA*(*a*, *b*),*LCA*(*a*, *b*, *c*)) ≥ ℓ^*^ and *depth*(*LCA*(*a*, *b*, *c*)) = *d* ≤ d^*^. Given our assumptions on the mutation process, the similarities *s*(*a*, *c*) and *s*(*b*, *c*) have the same distribution since *c* is the outgroup. Thus, we will focus on analyzing the quantity *E*[*s*(*a*, *b*) − *s*(*b*, *c*)] WLOG. Let *s*_*w*_(*u*, *v*) be the number of mutations shared by *u* and *v* that occurred after the point when the three lineages diverged from *LCA*(*u*, *v*, *w*). Then, we have that *s*(*a*, *b*) − *s*(*b*, *c*) = *s*_*c*_(*a*, *b*) − *s*_*a*_(*b*, *c*) since any mutation that occurred before *LCA*(*a*, *b*, *c*) is inherited by all three nodes and will contribute equally to *s*(*a*, *b*) and *s*(*b*, *c*). Let ℓ′ = dist(*LCA*(*a*, *b*),*LCA*(*a*, *b*, *c*)). *s*_*c*_(*a*, *b*) is distributed according to binomial(*k*, *p*), where *p*, the probability of a given character having the same mutation in *a* and *b*, satisfies

$$\begin{array}{cc} p= & \;e^{-\lambda d}\left(\left(1-e^{-\lambda \ell^{\prime }}\right)+e^{-\lambda \ell^{\prime }}{\left(1-d-e^{-\lambda (1-\ell^{\prime })}\right)}^{2}q\right)\\ & \geq e^{-\lambda d}\left(\left(1-e^{-\lambda \ell^{*}}\right)+e^{-\lambda \ell^{*}}{\left(1-e^{-\lambda (1-\ell^{*}-d)}\right)}^{2}q\right). \end{array}$$

Note that *e*^−*λd*^ is the probability that the mutation did not occur before *LCA*(*a*, *b*, *c*). The (1 − *e*^−*λ*ℓ′^) term is the probability that the shared mutation came from a single event that occurred on the path from *LCA*(*a*, *b*, *c*) to *LCA*(*a*, *b*), and the *e*^−*λ*ℓ′^(1 − *d* − *e*^−*λ*(1 − ℓ′)^)^2^*q* term is the probability that the shared mutation instead came from two independent events that happened on the path from the *LCA* to *a* and from the *LCA* to *b* independently (i.e., homoplasy). The second line follows from the fact that ℓ′ ≥ ℓ^*^.

Considering there are *k* characters, we then have:

$$\begin{array}{cc} E[s_{c}\left(a,b\right)]\geq & ke^{-\lambda d}(\left(1-e^{-\lambda \ell^{*}}\right)\\ & +e^{-\lambda \ell^{*}}{\left(1-e^{-\lambda (1-\ell^{*}-d)}\right)}^{2}q). \end{array}$$

In order for taxa *b* and *c* to share a particular mutation after their divergence from *LCA*(*a*, *b*, *c*) = *LCA*(*b*, *c*), the character must mutate on the path from the *LCA* to *b* and from the *LCA* to *c* independently (i.e., homoplasy). Thus, similar to the term above, we have

$$\begin{array}{c} E\left[s_{a}\left(b,c\right)\right]=ke^{-\lambda d}{\left(1-e^{-\lambda (1-d)}\right)}^{2}q. \end{array}$$

With the assumption that *λ*ℓ^*^ ≤ 1, we have that

$$\begin{array}{cc} E[s(a,b)- & s\left(b,c\right)]\geq ke^{-\lambda d}(1-e^{-\lambda \ell^{*}}\\ & +qe^{-\lambda \ell^{*}}\left(1-2e^{-\lambda (1-\ell^{*}-d)}+e^{-2\lambda (1-\ell^{*}-d)}\right)\\ & -q(1-2e^{-\lambda (1-d)}+e^{-2\lambda (1-d)}))\\ & =ke^{-\lambda d}(\left(1-e^{-\lambda \ell^{*}}\right)\left(1-q\right)\\ & +qe^{-2\lambda (1-d)}\left(e^{\lambda \ell^{*}}-1\right))\\ & \geq ke^{-\lambda d}\left(\left(1-e^{-\lambda \ell^{*}}\right)\left(1-q\right)+qe^{-2\lambda (1-d)}\lambda \ell^{*}\right)\\ & \geq 0.6k(e^{-\lambda d}\lambda \ell^{*}\left(1-q\right)+qe^{-\lambda (2-d)}\lambda \ell^{*})\\ & =0.6k\lambda \ell^{*}\left(e^{-\lambda d}\left(1-q\right)+qe^{-\lambda (2-d)}\right). \end{array}$$

The last inequality follows from the fact that 0.6*x* ≤ 1 − *e*^−*x*^ ≤ *x* for *x* ∈ [0, 1]. Let *δ*(*d*) = 0.6(*e*^−*λd*^ (1 − *q*) + *qe*^−*λ*(2 − *d*)^). We then have that for any triplet (*a*, *b*|*c*), where *depth*(*LCA*(*a*, *b*, *c*)) = *d* and *dist*(*LCA*(*a*, *b*, *c*), *LCA*(*a*, *b*)) ≥ ℓ^*^, the expected difference *s*(*a*, *b*) − *s*(*b*, *c*) satisfies

$$\begin{array}{c} E\left[s\left(a,b\right)-s\left(b,c\right)\right]\geq k\lambda \ell^{*}\delta \left(d\right). \end{array}$$

#### Defining a (ℓ^*^, d^*^)–oracle.

We are now ready to define the decision rule that will be used by the partial oracle. Let d^*^ ∈ [0, 1] be an arbitrary depth to which we expect the oracle to be correct, and let δ^*^ = min_*x* ∈ [0, d^*^]_*δ*(*x*). Notably, the δ^*^ function has a closed form that depends on *λ*, *q*, and d^*^ (*SI Appendix*, section A.1.1). For a particular triplet *a*, *b*, *c*, the oracle proceeds as follows:

(i) Set a threshold $t=\frac{1}{2}k\lambda \ell^{*}\delta^{*}$.
(ii) If there exists a pair *a*, *b* out of the triplet, such that *s*(*a*, *b*) − max(*s*(*a*, *c*),*s*(*b*, *c*)) > *t*, then return *c* as the outgroup. Otherwise, return *Null*.

In the following, we will prove that for a sufficiently large *k*, the function defined above is a (ℓ^*^, d^*^)−oracle. In particular, let (*a*, *b*|*c*) be any triplet where *LCA*(*a*, *b*, *c*) is at depth *d* < d^*^. We will prove that the following two conditions hold with high probability:

(i) If *dist*(*LCA*(*a*, *b*),*LCA*(*a*, *b*, *c*)) ≥ ℓ^*^, then *s*(*a*, *b*) − max(*s*(*b*, *c*),*s*(*a*, *c*)) > *t*
(ii) In all cases, min(*s*(*b*, *c*),*s*(*a*, *c*)) − *s*(*a*, *b*)< *t*.

To see that conditions (*i*) and (*ii*) imply correctness of the (ℓ^*^, d^*^)-oracle, note that the second condition guarantees that the oracle will not return the wrong answer when called on a triplet rooted at depth at most d^*^. It will therefore return either the correct outgroup or *Null*. The first condition guarantees that if the triplet is also separated by a path of length at least ℓ^*^, then the outgroup will be correctly returned.

*SI Appendix*, Fig. S1 provides empirical visualization of the (ℓ^*^, d^*^)-oracle using simulations. The simulations mimic the single-cell CRISPR-Cas9 lineage-tracing system and are described in *SI Appendix*, section A.3.1.

Before computing the *k* necessary to make conditions (*i*) and (*ii*) hold, we first state the following lemma which allows us to derive a worst-case bound on the probability of triplets failing to satisfy condition (*i*).

Lemma 1 (proof in *SI Appendix* , section A.1.2). *Let (a, b|c) be a triplet with α = dist(LCA(a, b, c),LCA(a, b)). P[s(a, b)−s(b, c)≥t] is increasing with α.*

Now, we can compute the *k* that ensures that both conditions (*i*) and (*ii*) are satisfied with high probability.

Lemma 2 (proof in *SI Appendix* , section A.1.3). Condition (i) holds with probability at least 1 − ζ if we have the following guarantees on the parameters q, λ, ℓ^*^, d^*^, and k:

$$\begin{array}{c} k\geq \frac{\left(96\log n+32\log1/\zeta \right)(\ell^{*}+\left(1-e^{-\lambda }\right)q)}{0.6\lambda \ell^{*2}\delta^{*}\left(1-q+qe^{-2\lambda }\right)}. \end{array}$$

*Both conditions *i* and *ii* hold with probability at least 1 − *ζ* if we have:*

$$\begin{array}{l} k\geq \max(\frac{(96\log n+32\log1/\zeta )q}{l^{*2}\delta^{*2}},\\ \;\;\;\;\;\frac{\left(96\log n+32\log1/\zeta )(l^{*}+(1-e^{-\lambda })q\right)}{0.6\lambda l^{*2}\delta^{*}(1-q+qe^{-2\lambda })}). \end{array}$$

**Fig. 2.**
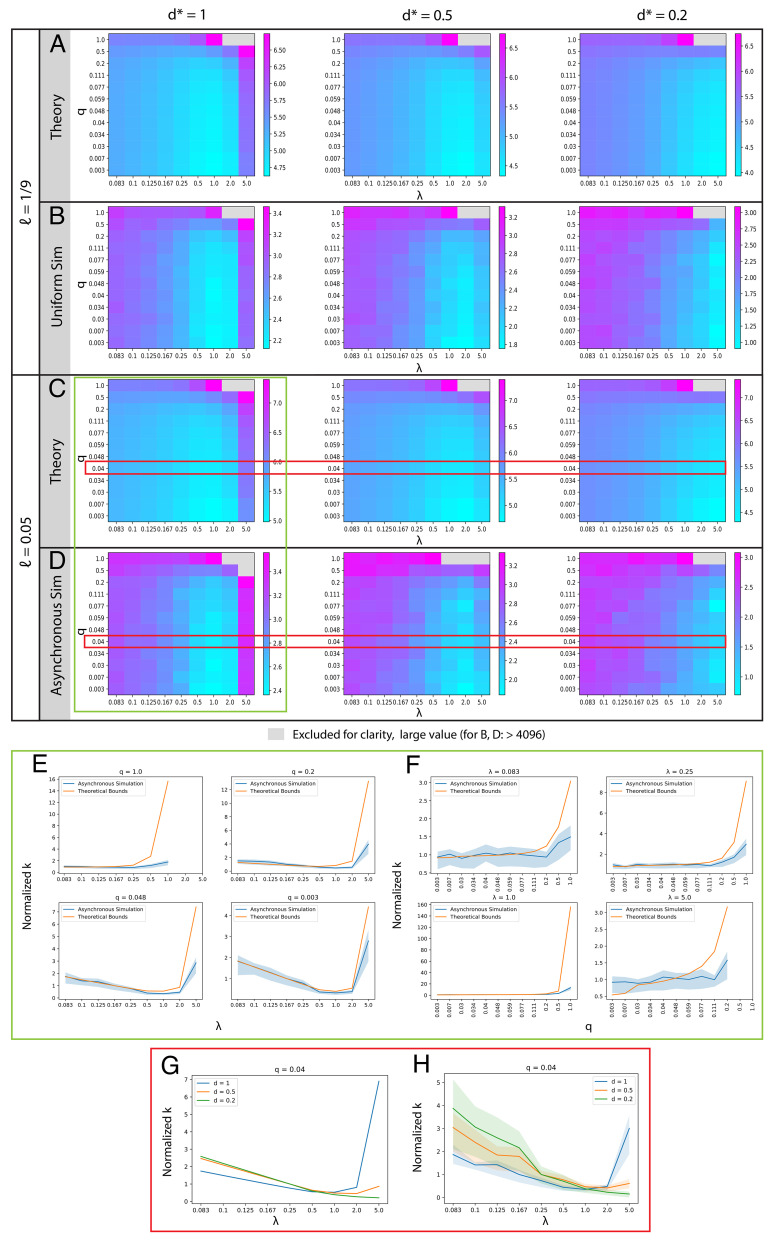
Comparing the threshold algorithm in theory and simulation. Simulated trees with 256 leaves, *n* = 256. (*A* and *C*) Theoretical sufficient lower bound on *k* required for 0.9 probability of exact reconstruction up to depth *d* for varying values of *d*, *q*, and *λ* for (*A*) ℓ = 1/9 and (*C*) ℓ = 0.05. (*B* and *D*) Minimum *k* required for 0.9 probability of exact reconstruction in simulation using a cell division topology with (*B*) uniform edge lengths with ℓ = 1/9 and with (*D*) an asynchronous cell division topology (*Materials and Methods*) with ℓ = 0.05. (*A*–*D*) Entries are *log*_10_ scaled. (*E* and *F*) Plots comparing the dependence of the minimum *k* on varying parameters in simulation with the theoretical bound (0.9 probability of exact reconstruction, ℓ = 0.05, d^*^ = 1). We report the dependence of *k* on (*E*) *λ* for fixed values of *q* and (*F*) *q* for fixed values of *λ* in simulations with asynchronous topologies. (*G*) Comparison of the dependence of the bound on *k* for 0.9 probability of exact reconstruction on *λ* for various values of *d*. (*H*) Comparison of the dependence of minimum *k* for 0.9 probability of exact reconstruction in the asynchronous simulation on *λ* for various values of *d*. (*E*, *F*, and *H*) For ease of comparison, the values of *k* are rescaled by the median value of *k* in each line. Point-wise 95% confidence intervals for the minimum *k* in simulation are generated from the regression coefficients using the delta method; *Materials and Methods*.

An empirical demonstration of the tightness of Lemma 2 using simulations with respect to *λ* and *q* is provided in Fig. 2 and with respect to *n* in *SI Appendix*, Fig. S3. The simulations are described in *Materials and Methods*.

#### Sufficient conditions for (ℓ^*^, d^*^)–oracle in the presence of missing data.

Now, we consider the possibility of missing data (dropout) and give several simple strategies to handle it. In our analysis, we consider two types of dropout events that may occur. A stochastic dropout is an event that occurs in and affects an individual cell (leaf), e.g., due to the limited sensitivity of single-cell RNA sequencing. A heritable dropout is an event that affects an entire clade, e.g., due to resection. Note that although we assume that dropouts occur independently in each character, dropouts observed in the same character at two different cells are not necessarily independent as they could have originated from the same heritable dropout event. Now, let *p*_*d*_ be the probability that a particular character of a particular cell suffers either heritable or stochastic dropout. Let (*a*, *b*|*c*) be an arbitrary triplet and let *ϵ* be the probability that no dropout occurred in a particular character in either *a*, *b*, or *c*. The probability that at least one cell of this triplet suffers a dropout at a particular character is maximized when the three cells share no common lineage and minimized when the three cells are phylogenetically proximal. We therefore have that (1 − *p*_*d*_)^3^ ≤ *ϵ* ≤ 1 − *p*_*d*_ (*SI Appendix*, section A.1.4). To account for this when revising our oracle definition, we now define *s*(*a*, *b*) as the number of characters shared by *a*, *b* that do not have dropout in *a*, *b*, or *c*. Note that in this case, the definition of *s*(*a*, *b*) depends on *c* but is well defined for every triplet *a*, *b*, *c*, so we can consider the same threshold-based triplet oracle before using this alternate similarity function. This means that for triplet (*a*, *b*|*c*),

$$\begin{array}{cc} E[s(a,b)-s(b,c)] & \geq k\epsilon \lambda \ell^{*}\delta \left(d\right)\geq k{\left(1-p_{d}\right)}^{3}\lambda \ell^{*}\delta \left(d\right)\\ E[s(b,c)] & \leq k\lambda^{2}\left(1-p_{d}\right)q. \end{array}$$

We can similarly define conditions (*i*) and (*ii*) with the threshold $t=\frac{1}{2}{\left(1-p_{d}\right)}^{3}k\lambda \ell^{*}\delta^{*}$ and see that if these conditions hold, then we have an (ℓ^*^, d^*^) partial oracle. We can also apply the same Chernoff bounds from the previous sections to get the following conditions on *k* to ensure that conditions (*i*) and (*ii*) hold.

Lemma 3 (proof in *SI Appendix* , section A.1.4). In the presence of missing data at a rate of *p*_*d*_, condition (*i*) holds with probability at least 1 − *ζ* if we have the following guarantees on the parameters *q*, *λ*, ℓ^*^, d^*^, and *k*:

$$\begin{array}{c} k\geq \frac{\left(96\log n+32\log1/\zeta \right)(\ell^{*}+\left(1-e^{-\lambda }\right)q)}{0.6\lambda \ell^{*2}\delta^{*}{\left(1-p_{d}\right)}^{3}\left(1-q+qe^{-2\lambda }\right)}. \end{array}$$

*Both conditions (*i*) and (*ii*) hold with probability at least 1 − *ζ* if we have:*

$$\begin{array}{l} k\geq \max(\frac{(96\log n+32\log1/\zeta )q}{l^{*2}\delta^{*2}{\left(1-p_{d}\right)}^{5}},\\ \;\;\;\;\;\;\;\frac{\left(96\log n+32\log1/\zeta )(l^{*}+(1-e^{-\lambda })q\right)}{0.6\lambda l^{*2}\delta^{*}{\left(1-p_{d}\right)}^{3}(1-q+qe^{-2\lambda })}). \end{array}$$

We provide an alternative analysis in the case where only stochastic dropout is present in *SI Appendix*, section A.2.1, with empirical demonstration of the tightness of that analysis via simulations in *SI Appendix*, Fig. S4 and section A.3.4.

#### Top–down algorithms for oracle-based partial tree inference.

Given our results on the correctness of the (ℓ^*^, d^*^)−oracle, we are now ready to define the respective algorithm. Assuming ℓ−bounded edge lengths in the ground truth tree, we use an oracle in which ℓ^*^ is set to ℓ. With that (ℓ,d^*^)−oracle, the algorithm guarantees accurate reconstruction up to depth d^*^ when given a sufficiently large *k*.

Theorem 1. *In the lineage-tracing regime, if*

$$\begin{array}{c} k\geq \frac{\left(96\log n+32\log1/\zeta \right)(\ell +\left(1-e^{-\lambda }\right)q)}{0.6\lambda \ell^{2}\delta^{*}\left(1-q+qe^{-2\lambda }\right)}, \end{array}$$

and all edges in 𝒯 at depth at most d^*^ have length at least ℓ, there exist polynomial time algorithms that return a tree which correctly resolves all triplets whose LCA is at depth at most d^*^ with probability at least 1 − *ζ*.

**Proof of Theorem 1.** By taking ℓ^*^ = ℓ, condition (*i*) will hold for all triplets (*a*, *b*|*c*) whose LCAs are at depth at most d^*^ on 𝒯 as *dist*(*LCA*(*a*, *b*, *c*),*LCA*(*a*, *b*)) ≥ ℓ for all triplets. The above bound on *k*, by lemma 2, implies that condition (*i*) is satisfied with probability 1 − *ζ*. It then suffices to show that whenever condition (*i*) is satisfied, there exists a polynomial time algorithm that constructs a tree which correctly resolves all triplets whose LCAs are at depth at most d^*^. We present a simple top–down recursive splitting algorithm which is guaranteed to return correct splits up to depth d^*^. This algorithm has a runtime of *O*(*kn*^2^) for each recursive call, where *n* is the size of the input to the call.

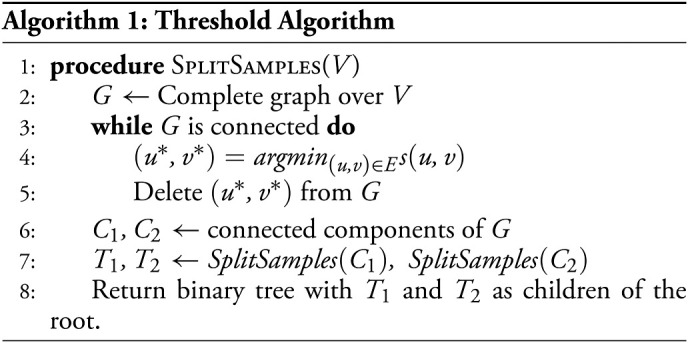

To prove correctness, let *V* be the set of samples at some recursive call in the algorithm. Let *r*′ be the LCA of *V* in 𝒯 at depth *d* ≤ d^*^. Let *L* and *R* be the samples in the left and right subtrees of *r*′, respectively. Let *a* ∈ *L* and *b* ∈ *R* be arbitrary. By condition (*i*), we know that for any *c* ∈ *L*, *s*(*a*, *c*)> *s*(*a*, *b*) and for any *c*′∈*R*, *s*(*b*, *c*′) > *s*(*a*, *b*). This means that whenever (*a*, *b*) is in the graph, and right after (*a*, *b*) is deleted from the graph if it ever happens, all neighbors of *a* and *b* will remain connected to them. Thus, the graph must remain connected until all edges in the cut *L*|*R* are deleted, and *L* and *R* will still remain connected immediately after all cut edges are deleted, giving us the correct split. This means that the algorithm will keep returning correct splits as long as the LCA of all samples in a recursive call has depth at most d^*^.

**Proof of Corollaries 1 and 2.** If we take *λ* and *q* as constants and d^*^ = 1, Theorem 1 implies that it is asymptotically sufficient to have $k=O(\frac{\log n}{\ell^{2}})$ in order to ensure exact recovery of the entire ground truth tree with high probability. When dropouts are taken into account in the general case, the bound becomes $k=O(\frac{\log n}{\ell^{2}{\left(1-p_{d}\right)}^{3}})$ by Lemma 3 (*SI Appendix*, section A.1.4) because 1/(1 − *p*_*d*_)^3^ factor is needed to ensure that condition (*i*) still holds. Notably, when only stochastic dropouts are considered, the bound becomes $k=O(\frac{\log n}{\ell^{2}{\left(1-p_{d}\right)}^{2}})$ by lemma 9 (*SI Appendix*, section A.2.1).

In the next theorem, we see that when we do not have a lower bound on edge lengths, we can still construct a tree that correctly resolves all triplets that are well separated.

Theorem 2. *In the lineage-tracing regime, if the number of characters satisfies*

$$\begin{array}{l} k\geq \max(\frac{(96\log n+32\log1/\zeta )q}{l^{*2}\delta^{*2}},\\ \;\;\;\;\;\;\;\frac{\left(96\log n+32\log1/\zeta )(l^{*}+(1-e^{-\lambda })q\right)}{0.6\lambda l^{*2}\delta^{*}(1-q+qe^{-2\lambda })}), \end{array}$$

and ℓ^*^ is an arbitrary parameter, then there exist polynomial time algorithms that return a tree which correctly resolves all triplets, (*a*, *b*|*c*) such that *dist*(*LCA*(*a*, *b*, *c*),*LCA*(*a*, *b*) ≥ ℓ^*^ and at *depth*(*LCA*(*a*, *b*, *c*)) ≤ d^*^ with probability at least 1 − *ζ*.

**Proof of Theorem 2.** Again, by lemma 2, it suffices to show that if conditions (*i*) and (*ii*) both hold, then there exists an algorithm which resolves all triplets, (*a*, *b*|*c*) such that *dist*(*LCA*(*a*, *b*, *c*),*LCA*(*a*, *b*)≥ℓ^*^, and *depth*(*LCA*(*a*, *b*, *c*)) ≤ d^*^. We can apply the classical Aho’s algorithm to recover a tree that is consistent with all triplets resolved by the (ℓ^*^, d^*^)−oracle, which is guaranteed to us by conditions (*i*) and (*ii*). The algorithm is specified here for completeness; other supertree algorithms can be used as well.

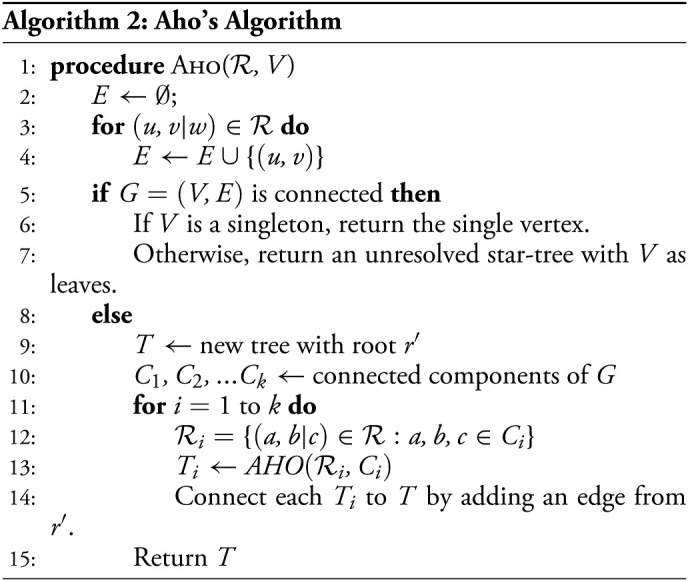

Let ℛ be the set of triplets for which we received a non-*NULL* answer from the oracle, and let *V* be the set of leaf nodes. Note that ℛ must include all triplets that are at depth at most d^*^ and whose internal nodes are separated by a path of length at least ℓ^*^, since they satisfy condition (*i*). It may also include incorrect triplets that are of depth more than d^*^.

To prove the correctness of the algorithm, we must show that if (*a*, *b*|*c*) is a triplet in the ground truth tree with *depth*(*LCA*(*a*, *b*, *c*)) ≤ d^*^ and *dist*(*LCA*(*a*, *b*),*LCA*(*a*, *b*, *c*)) ≥ ℓ^*^, then (*a*, *b*|*c*) is correctly resolved in the tree returned by the algorithm. First, assume by contradiction that the triplet is represented wrongly (WLOG) as (*a*, *c*|*b*) in the returned tree. The presence of a wrong triplet (*a*, *c*|*b*) in the returned tree means that at some point, there was a recursive call on a set of leaves *a*, *b*, *c* ∈ *V* such that *a*, *c* were in a connected component of *G* not containing *b*. However, condition (*i*), combined with the assumption that *dist*(*LCA*(*a*, *b*),*LCA*(*a*, *b*, *c*)) ≥ ℓ^*^, implies that if *a*, *b*, *c* ∈ *V*, then there is an edge between *a* and *b*, which means that *b* must be in the same connected component as *a* and *c*.

Next, assume by contradiction that *a*, *b*, *c* all have the same parent in the tree returned by the algorithm. First, note that this cannot happen in line 15. This follows trivially since by definition *a* and *b* are initially in the same connected component. Therefore, the only way a trifurcation can happen is if a connected component that contains *a*, *b*, and *c* is split into three or more components (with *a*, *b*, and *c* on different components), each sent to a separate recursive call (line 14). This cannot happen since the presence of *a*, *b*, and *c* entails the inclusion of an edge between *a* and *b* in that component (per line 4). This means that there was a recursive call on a set of leaves *a*, *b*, *c* ∈ *V* such that the connectivity graph *G* over *V* is connected (i.e., the algorithm reached step 8). Let *r*′ be the LCA of all vertices of *V* in 𝒯. Let *L* and *R* be the vertices in *V* descended from the left and right children of *r*′, respectively. Since *depth*(*r*′) < d^*^ condition (*ii*) implies that any triplet with all leaves in *V* will be either correctly classified or assigned *Null* by the oracle. But, there are no edges between *L* and *R*, which means that *V* is not connected, thus arriving at a contradiction once again. Thus, the only possibility is for (*a*, *b*|*c*) to be correctly classified in the inferred tree.

**Proof of Corollary 3.** We take *λ* and *q* as constants and d^*^ = 1. Additionally, we make no lower-bound assumptions on the edge length ℓ and take ℓ^*^ to be an arbitrary parameter. Theorem 2 then implies that it is asymptotically sufficient to have $k=O(\frac{\log n}{\ell^{*2}})$ in order to ensure exact recovery of all triplets (*a*, *b*|*c*) such that *dist*(*LCA*(*a*, *b*, *c*),*LCA*(*a*, *b*)) ≥ ℓ^*^ with high probability.

### Simulations for the Threshold Algorithm.

Theorem 1 gives a lower bound on the number of characters *k* sufficient for exact phylogenetic reconstruction in the case where there is a minimum edge length ℓ. In order to study whether the theoretical asymptotic relationships between experimental parameters are observed in the empirical case as well as get a better sense for the number of characters that may be necessary in practice (a number that may be lower than our estimate of sufficiency), we turn to simulations. Specifically, we explore the empirical minimum *k* necessary for exact reconstruction (all triplets resolved correctly) up to some depth d^*^ and as a function of the state collision probability *q* and the mutation rate *λ* over sampled tree topologies (*Materials and Methods* for description of simulations).

First, we visualize the dependence of *k* on the other parameters in the bound of Theorem 1. Fig. 2 *A* and *C* depict the theoretical dependence of *k* sufficient for high probability (0.9) of exact reconstruction on varying *λ* and *q* for $\ell =\frac{1}{9}$ or 0.05. We observe that in the regions where *q* > ℓ/*λ*, the sufficient *k* increases sharply. This is since for smaller values of *q*, the asymptotic requirement for *k* becomes $O(\frac{log(n)}{\ell })$, whereas for higher values of *q*, we get the general result of $O(\frac{log(n)}{\ell^{2}})$. Additionally, we observe interesting behaviors regarding *λ*. In particular, when d^*^ is large enough (requiring exact reconstruction of the entire ground truth phylogeny or its top half), both excessively small and large values of *λ* lead to a larger requirement for *k*. Intuitively, this is due to the lack of mutations or due to mutation saturation, in both cases leading to less informative input (Fig. 2*H*). When the goal becomes partial reconstruction of only the top (20%) of the phylogeny and d^*^ is small, *k* no longer increases with large *λ* in our explored range. This is because δ^*^ in the denominator of the bound shifts from *e*^−*λ*^ to 1 as d^*^ → 0. Intuitively, toward the top of the phylogeny, characters are yet to be saturated, allowing cells whose LCAs are near the top of the tree to be resolved. This suggests that saturation is less problematic if only distal relationships need to be resolved correctly, i.e., in the case of d^*^ ≪ 1.

In order to test the performance of the threshold algorithm in realistic empirical settings, we simulate CRISPR-Cas9-induced phylogenies over two topological regimes: one with uniform edge lengths separating cell divisions ($\ell =\frac{1}{9}$) and one with asynchronous cell division (ℓ = 0.05) described in *Materials and Methods* (Fig. 2 *B* and *D*). The first regime aims to mimic a cell division process that has a regular molecular clock, i.e., with edge lengths bounded both from above and below. The second regime is meant to mimic a more general stochastic cell division process that only has a minimum bound on edge length.

In comparing the trends in the dependence of *k* on *λ* and *q*, we find that the theoretical analysis and the simulations are consistent both in terms of the direction dependence on the parameters and on the inflection points at which the minimal *k* increases more rapidly. The largest discrepancies in the trends occur in the regions in which *λq* is high. In these regions, the empirical increase in *k* is not nearly as sharp as the theoretical bound suggests. Hence, the theoretical bound overestimates *k* relative to other values in these regions. Additionally, we observe that as d^*^ decreases, the empirical *k* decreases (Fig. 2*I*). This last result matches the trend in the bounds regarding d^*^. Ultimately, the theoretical estimate predicts the empirical trends well; however, we do find that the absolute number of necessary characters (as found by the simulation) is much lower than the theoretical estimate.

### Upper-Bounded Edge Lengths and Bottom–Up Approaches.

In the previous sections, we saw that when *λ* and *q* are fixed to be a constant, the number of characters needed for exact recovery with high probability is $O(\frac{\log n}{\ell^{2}})$, where ℓ is the minimum edge length. It can also be shown that if we are able to bound *λ* and *q* such that *λq* ≤ ℓ, then the bound becomes tighter: $k=O(\frac{\log n}{\ell })$. However, while *λ* can be controlled experimentally by calibrating the affinity of the lineage tracer’s guide RNAs, there is currently no way to control *q*—a quantity which relies on the endogenous DNA repair process.

In order to achieve this tighter bound without direct dependence on *q*, we instead use an additional assumption on ℓ—namely that there is some maximal (in addition to a minimal) possible period of time between the birth of a given node and the birth of its parent. If our set of leaves includes all the cells in the phylogeny, then this translates to an upper bound on the time between cell divisions. In the more common scenario of sampling only a small subset of cells from a given clone, each edge in the ground truth tree can correspond to a series of cell division events. However, in either case, a strict upper bound on the length always exists, corresponding to the duration of the lineage-tracing experiment. In the following section, we show that with such an upper bound on edge length, we can achieve exact recovery with high probability when $k=O(\frac{\log n}{\ell })$, provided an upper bound on the probability of the most likely mutation (max_*j*_(*q*_*j*_)) and on *λq*. The latter bound can be less strict than ℓ, depending on the ratio between the lengths of the longest and shortest edges. More importantly, under these revised assumptions, we can achieve a bound on the minimal required *k* that is independent of the value of *q*.

Theorem 3 *(proof in *SI Appendix* , section A.1.5).* *Let 𝒯 be a tree of height 1 over *n* leaves. Let ℓ and *c* be constants such that each edge (*u*, *v*) ∈ 𝒯 has* $\ell \leq l\left(u,v\right)\leq \sqrt{c\ell }$. *Suppose that for each character we have* $q_{\max}:=\max_{j}\left(q_{j}\right)\leq \frac{3}{16(1-e^{-\lambda \sqrt{c\ell }})}$ *and* $\lambda q<\frac{\beta }{\max(1,c)}$, *where* $\beta <\frac{1}{1+C+2{\left(e^{-\lambda \ell }+2\lambda \sqrt{c\ell }q_{\max}\right)}^{2}}$ *and* $C\;=\;2\sqrt{c\ell }e^{-\lambda }+4\lambda c\ell q_{\max}$. *Then, there exists an algorithm that, with high probability, will recover 𝒯 if the number of characters, *k*, is at least*

$$\begin{array}{c} \frac{20\log n+10\log\left(1/\zeta \right)}{\lambda e^{-\lambda }\ell \left(1-\beta \left(1+C\right)\right)(1-\beta \left(1+C\right)-2\beta {\left(e^{-\lambda \ell }+2\lambda \sqrt{c\ell }q_{\max}\right)}^{2})}. \end{array}$$

If we take the limit as ℓ → 0 or *n* → ∞, we get the following result:

Corollary 5 *Let 𝒯 be a tree of height 1 over a sufficiently large number of leaves *n*. Define ℓ, *c*, and *q*_max_ as in Theorem 3 with similar bounds. If* $\lambda q\leq \frac{\beta }{\max(1,c)}$, *where* $\beta <\frac{1}{3}$, *then there exists an algorithm that can, with high probability, recover 𝒯 if the number of characters satisfies:*

$$k\geq \frac{20\log n+10\log\left(1/\zeta \right)}{\lambda e^{-\lambda }\ell \left(1-\beta \right)\left(1-3\beta \right)}.$$

Note that the bound on *β* is not the tightest possible, and it was chosen to simplify calculations. Additionally, we present an alternative analysis in *SI Appendix*, section A.2.2 (Theorem 4) that yields a bound which has a looser constraint on the *λ* and *q* parameters as ℓ tends to 0.

Consider the following greedy algorithm which iteratively joins partially constructed subtrees by picking the pair with the most similar roots and then joining them by inferring a new root by maximum parsimony. Let *S* denote the set of subtrees at any particular iteration. Let *T* ∈ *S* denote an inferred subtree, and let *r*(*T*) denote the root of that subtree. Let *r*(*T*)_*i*_ denote the state of the inferred *i*^*th*^ character of *r*(*T*).

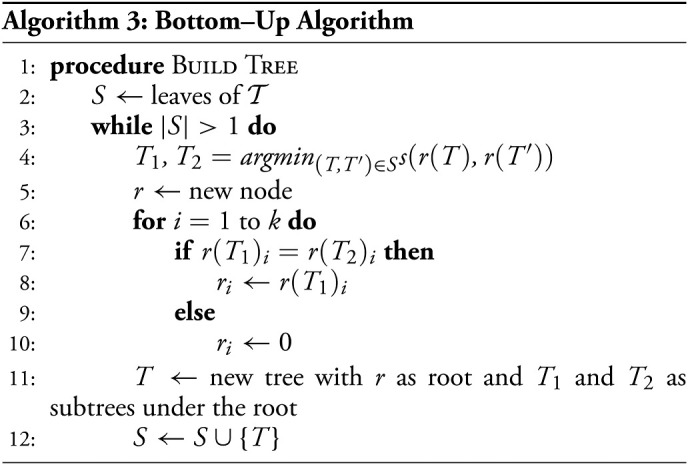

We note that this algorithm has been presented in Sugino et al. (35), although no theoretical guarantees on accuracy are given in that work.

**Fig. 3.**
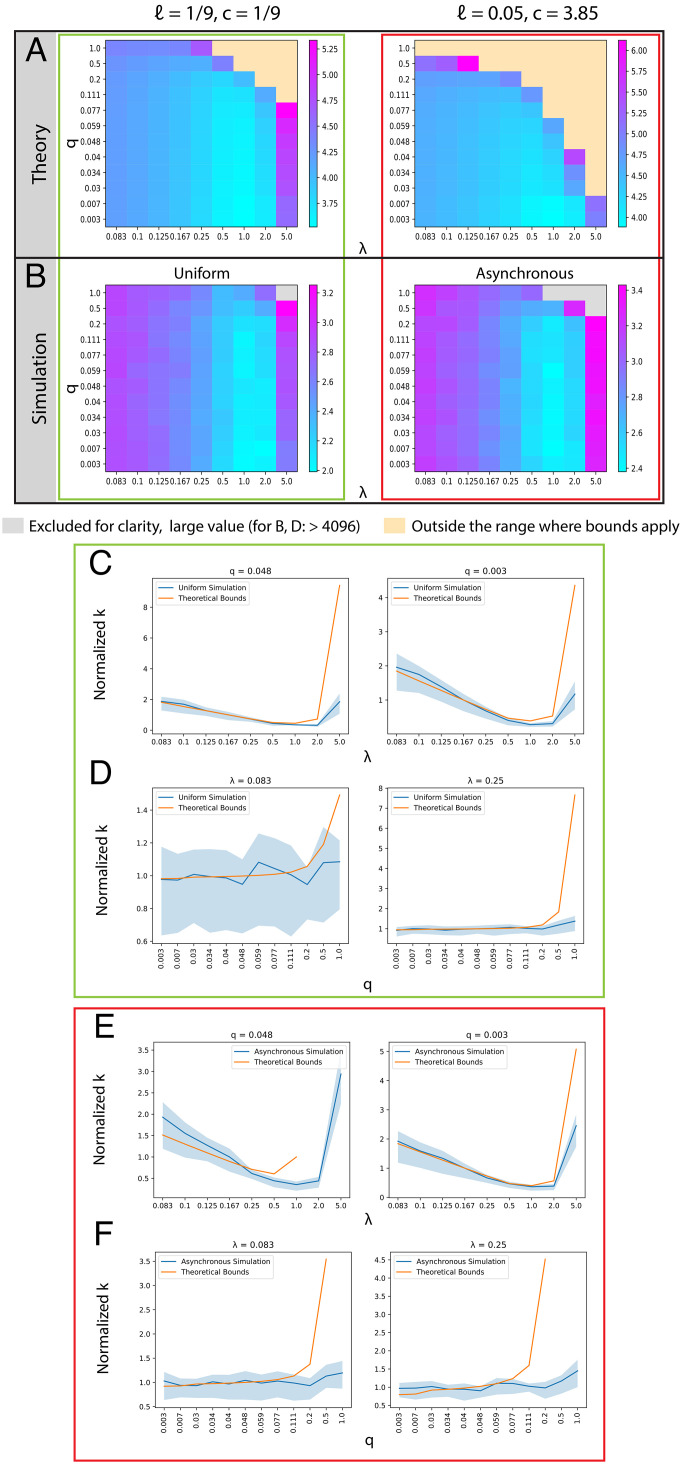
Comparing the bottom–up algorithm in theory and simulation. Simulated trees with 256 leaves, *n* = 256. (*A* and *B*) Entries are *log*_10_-scaled. (*A*) Theoretical sufficient lower bound on *k* required for 0.9 probability of exact reconstruction on varying values of *q* and *λ*, taking *β* = *λq*max(1, *c*). As the state distributions are uniform, *q*_*j*_ = *q* for each value of *q*. Left: ℓ = 1/9 and *c* = 1/9 for comparison with the simulation using a cell division topology with uniform branch lengths. Right: ℓ = 0.05 and *c* ≈ 3.85 such that > 99.99% of branch lengths in the simulation using an asynchronous cell division topology (description in Materials and Methods) fall within the upper bound. (*B*) Minimum *k* required for 0.9 probability of perfect tree reconstruction in simulations, Left, Uniform topology, ℓ = 1/9. Right, Asynchronous topology, ℓ = 0.05. (*C*–*F*) Plots comparing the dependence of the minimum *k* on varying parameters in simulation with the theoretical lower bound (0.9 probability of exact reconstruction). We report the dependence on (*C* and *E*) *λ* for fixed values of *q* and on (*D* and *F*) *q* for fixed values of *λ*, in simulations with uniform edge lengths (*C* and *D*) and with asynchronous topologies (*E* and *F*). (*C*–*F*) For ease of comparison, the values of *k* are rescaled by the median value of *k* in each line. Point-wise 95% confidence intervals for the minimum *k* in simulation are generated from the regression coefficients using the delta method, *Materials and Methods*.

In *SI Appendix*, section A.1.5, we prove that under the conditions in Theorem 3, this algorithm correctly returns the ground truth tree 𝒯 with high probability. An empirical demonstration of the tightness of Theorem 3 using simulations with respect to *λ* and *q* is provided in Fig. 3 and with respect to *n* in *SI Appendix*, Fig. S3. The simulations are described in *Materials and Methods*.

**Proof of Corollaries 4 and 5.** The bottom–up algorithm shows that there exists a polynomial time algorithm that ensures that $k=O(\frac{\log n}{\ell })$ characters is sufficient asymptotically for exact recovery of the tree. As *n* → ∞, we get that ℓ → 0 and *C* → 0. With these, we get that $\beta <\frac{1}{3}$. With the simpler bound on *β*, we also get that $k=O(\frac{\log n}{\ell })$ characters are sufficient asymptotically for exact recovery of the tree.

### Simulations for the Bottom–Up Algorithm.

As with the threshold algorithm, for the bottom–up algorithm, we use simulations to study whether the relationships between experimental parameters in the bound of Theorem 3 are preserved empirically as well as to get a better sense of the empirical necessary number of characters.

As above, we first visualize the dependence of *k* on the other parameters in the bound of Theorem 3. Fig. 3*A* visualizes the theoretical bound for *k* sufficient for high probability (0.9) of exact reconstruction across varying values of *λ* and *q*. We consider two regimes: one with ℓ = 1/9, *c* = 1/9 and one with ℓ = 0.05, *c* ≈ 3.85. Since *q* does not explicitly appear in the bound for *k*, we instead use it to define a value for *β*, using its lower bound: *β* := *λq* ⋅ *max*(1, *c*). Plugging this in provides a lower bound for the sufficient *k*, which we plot here. Regions where the lower bound on *β* becomes larger than its upper-bound requirement (per Theorem 3) are excluded. From this figure, it can be seen that the dependence of *k* on *λ* is similar in Theorem 3 as to Theorem 1. That is, *k* increases significantly for both excessively small and large values of *λ*. However, there is a contrast in the dependence of *k* on *q*. Although in the bound for Theorem 3, *k* does increase with *q* through the dependence of *β*, *k* is not as sensitive to large values of *q* as in Theorem 1. Further, as the bound is quadratic in $\frac{1}{1-\beta }$, the *k* increases rapidly with respect to *β* := *λq* ⋅ *max*(1, *c*).

We tested the bottom–up algorithm in the same simulation regimes (same tree and lineage-tracing parameters) as the threshold algorithm (Fig. 3*B*). Concordant with the theoretical results, we observed that the minimum required *k* is less sensitive to *q*, compared with the threshold algorithm. Furthermore, in both results, we see similar trends in dependence on *λ* (Fig. 3 *C* and *E*) and *q* (Fig. 3 *D* and *F*). The main discrepancy between the theory and the simulation occurs where *β* := *λq* ⋅ *max*(1, *c*) approaches our upper bound for *β* (i.e., the values that border the regions that were excluded from Fig. 3*A*). In those cases, we see that the theoretical bound is looser and overestimates *k* relative to the simulations.

As in the simulations for the threshold algorithm, these simulations show that the relationships observed in the asymptotic trends of the dependence of *k* on *λ* and *q* still hold in the empirical case, and they also give tighter empirical conditions on the necessary *k* for exact reconstruction. We observe that the empirical minimum necessary *k* for the bottom–up algorithm is overall lower than that of the threshold algorithm in the uniform simulation but is comparable in the asynchronous simulation with a high value of *c* (Fig. 3 *A* and *B*, *Right*, and Fig. 3 *E* and *F*). These results suggest that the bottom–up algorithm can achieve exact reconstruction with fewer characters empirically than the threshold algorithm, but it requires the variance in the division times of the ground truth phylogeny to be small.

## Discussion

In this paper, we have established sufficient conditions for a high probability of exact reconstruction of the ground truth phylogeny in the single-cell CRISPR-Cas9 lineage-tracing setting. These guarantees show that despite complications with the lineage-tracing process such as homoplasy, missing data, and lack of mutation information, exact reconstruction can still be achieved given sufficient information capacity in the experiment (as measured by the number of recording sites). In addition to showing the feasibility of exact reconstruction, these theoretical results relate the difficulty of the reconstruction problem in the number of sufficient characters to the experimental parameters. We anticipate that these results can inform researchers as to how to reduce the number of necessary characters or best aid downstream reconstruction of the phylogeny given the available number of characters through careful engineering of single-cell CRISPR-Cas9 lineage-tracing experiments.

Although having more characters is preferable, we recognize that currently there are practical limits on the number of recording sites that can be incorporated into CRISPR-Cas9 systems, with current systems offering only on the scale of tens of recording sites (1–6, 8, 24). The limitations in the number of characters motivate the optimization of the other experimental parameters. We show that for exact reconstruction, a mutation rate that is neither too high nor low gives the best results, congruent with the intuition that a middling rate balances mutation saturation and mutation-less edges. Additionally, we formalize the intuition that a state distribution with a low rate of collision *q* makes the reconstruction problem easier in avoiding homoplasy. We believe though that in designing lineage-tracing systems, effort is better placed on carefully engineering the Cas-9 cutting rate than optimizing the state distribution. This is for two reasons that are apparent from our bounds and simulations: 1) Current CRISPR-Cas9 lineage-tracing systems are capable of generating indel distributions with thousands of possible indels and *q*s that lie outside of the range where *k* explodes with *q* (5, 6, 8, 9) and 2) further decreasing *q* has diminishing returns on *k*. Finally, we show the difficulty that missing data pose for the problem of exact reconstruction. Using the presented methods, the number of additional characters needed to overcome missing data is cubic (quadratic in the case of only stochastic missing data) in $\frac{1}{1-p_{d}}$, where *p*_*d*_ is the probability of missing data. Ultimately though, we see in our simulations that even under favorable experimental parameters, the current number of characters is far insufficient for exact reconstruction, especially considering the considerable amounts of missing data and the large number of samples (*n*) that we see in real single-cell CRISPR-Cas9 lineage-tracing experiments. We thus challenge the field to develop systems that allow for considerably more characters.

Another key result is that the (ℓ^*^, d^*^)-oracle in the top–down algorithms allows researchers to tailor the granularity of their reconstruction accuracy to what is achievable given the number of available characters. We show that substantially fewer characters are required if one is interested only in correctly resolving triplets that diverged early in the tree (small d^*^) and well-separated triplets (large ℓ^*^, regardless of the true minimum edge length ℓ). The choice of ℓ^*^ and d^*^ is often motivated by the biological questions at hand. For example, one could be particularly interested in studying the differentiation and restriction events that occur before and up to the exit of pluripotency in mammalian development, occurring around embryonic day 6.5 in mice. In the study of Chan et al. (5), cells were traced until around embryonic day 9, and thus, d^*^ could be set to 6.5/9 ≈ 0.722 in a proposed lineage-tracing study. One could also choose an ℓ^*^ that is larger than the minimum edge length ℓ. For example, in *Xenopus laevis*, the cell cycle time during the cleavage phase is ≈ 20 min and ≈4 h during gastrulation (36). If one is most interested in resolving developmental relationships during gastrulation, one could set ℓ^*^ to be 4 h (normalized by total time) instead of 20 min.

In addition to motivating the design of single-cell CRISPR-Cas9 lineage-tracing experiments, our model motivates theoretical and algorithmic development for these systems. The sufficient bounds that we reach in our asymptotic analyses are not tight, as demonstrated by simulation, but we believe that these bounds can likely be further improved to give a better sense of the necessary *k* analytically. Future approaches may extend our model to better capture aspects of current lineage-tracing technologies or account for new technological developments. For example, in our analysis, we assume that the mutation rate *λ* is constant throughout the entire phylogeny and across recording sites. However, modeling nonconstant mutation rates over time could allow one to better capture the effects of diminishing mutation rates due to reductions in the expression of Cas9 components, and modeling characters with different rates may better capture the effects of engineering distinct target sites with different mutation rates. Technologies in the future could also leverage such strategies to improve reconstruction accuracy by increasing recording resolution while avoiding mutation saturation. We also assume that the characters mutate and acquire missing data independently, although indels and missing data events can span multiple recording sites. Future approaches could take advantage of the structure present in these multisite events. Finally, although our analysis handles missing data by ignoring missing characters, the structure of heritable data offers additional information that could be better leveraged (i.e., utilized in the same way as any other mutation). The challenge, naturally, is to distinguish between the two types of missing data.

Here, we perform a theoretical analysis of single-cell CRISPR-Cas9 phylogenetic reconstruction. In doing so, we have developed a generative model for this type of data, which we hope will frame future analysis of single-cell CRISPR-Cas9 lineage-tracing systems akin to the Jukes–Cantor model in other molecular phylogenetic studies. With this theoretical framework and the accompanying algorithms, our work naturally complements recent efforts to develop and understand algorithms for this type of data (31). Ultimately, we believe that this work will continue to inform and orient both algorithmic and experimental methods as the technology and field evolve.

## Materials and Methods

Simulations and algorithms are implemented in Python in the Cassiopeia software suite (6) (https://github.com/YosefLab/Cassiopeia). The analyses utilized the NetworkX package (37). Additional implementation specifics are provided in *SI Appendix*, section A.4.

### Simulating Lineage-Tracing Experiments.

In our simulations, we simulated forward-time lineage-tracing experiments using our generative model. We split the simulation into two steps.

#### Simulating cell division topologies.

First, we simulate a continuous-time, binary, symmetric cell division topology. Then, we simulate single-cell CRISPR-Cas9 lineage-tracing data over the given topology. The end result is a phylogenetic tree representing the single-cell lineage-tracing experiment. The tree topology also records the ground truth phylogenetic relationships between the observed cells.

We begin by describing the two simulation schemes used to generate the tree topology. The first scheme simulates a cell division regime with regular cell division (uniform edge lengths). We start with a complete binary tree and add an implicit root, attaching this root to the root of the complete binary tree by an edge. The edge represents the lifetime of the root along which mutations can be acquired. For all figures besides *SI Appendix*, Fig. S3, we generated trees with 256 leaves representing cells observed at the end of the experiment. For *SI Appendix*, Fig. S3, we generated trees of various sizes, exponentially increasing *n*. Each edge on the phylogeny has length $\frac{1}{log_{2}\left(n\right)+1}$ as the path from the implicit root to each leaf has *log*_2_(*n*)+1 edges and the length of the experiment is normalized to 1.

The second simulation scheme represents an asynchronous cell division regime, with stochastic waiting times between cell divisions and cell death. We model a forward-time Bellman–Harris model with extinction (38). This generalizes the birth–death process (39), a commonly used phylogenetic model, such that the distribution of waiting times between division and death events is arbitrary. In our case, waiting times between division events are distributed according to an exponential distribution that is shifted by a constant *a* = 0.05, representing the minimum time between cell division events. The distribution of death waiting times is exponential as we assume that cell death does not have a minimum time. The stopping condition is when all lineages reach time = 1, meaning that each lineage will have total path length from the root of 1. Thus, the number of leaves is stochastic, so to control for the number of leaves, we take only trees that have between 205 and 307 leaves. Note that these trees also have a singleton edge coming from the root.

#### Simulating CRISPR-Cas9 lineage-tracing data.

Given a tree topology, we simulate a CRISPR-Cas9 mutagenesis process over it. Along each edge with length *t*, independently for each character, we simulate the probability that a mutation will occur as 1 − *e*^*λt*^. If a character has been chosen to mutate, we then draw from the state distribution to determine the state the character acquires. In this case, this is a uniform distribution with *m* = 1/*q* states (note that in the uniform case, $q=\sum_{j=1}^{m}{\frac{1}{m}}^{2}=1/m$). Once a mutation is acquired on an edge, that mutation persists in all downstream nodes. Then, the set of mutations acquired along the path from the implicit root to each leaf node is recorded, which then forms the character information for all observed cells. If the simulation involves missing data, then *p*_*s*_ proportion of characters are uniformly at randomly changed to a state representing missing data (−1). This character information is the input to the reconstruction algorithm.

### Simulating the Minimum Necessary k for Each Algorithm.

Here, we describe the process by which we determined the minimum *k* necessary for 90% probability of a given reconstruction criterion in our simulations: either full reconstruction, partial reconstruction for triplets whose LCA is up to depth *d*, or triplets correct (*SI Appendix*, Fig. S5 and section A.3.5). For a given value of *k* and a given set of parameters, we verify whether it is sufficient for 90% probability of a given scoring criterion. First, we simulate 10 lineage-tracing trees as follows: In the uniform simulation, we overlay 10 mutation datasets over the complete binary topology with uniform edge lengths, and in the asynchronous simulation, we simulate 10 different topologies and overlay 1 mutation dataset per topology. Then, we reconstruct each tree from its observed cells (leaves) using the relevant algorithm and compare the corresponding reconstructed tree to each ground truth tree. If ≥ 9 out of those 10 trees meet a scoring criterion, then we say that this *k* is sufficient.

To efficiently explore the space of *k*, we first exponentially (base 2) increase the value of *k* until a sufficient *k* is found or a maximum value is reached (4,096 in the case of no missing data and 8,192 in the case of missing data). Once we find a sufficient *k*, we perform a binary search in the bin between that value and half that value. Finally, we record the number of trees correctly reconstructed out of 10 for each value of *k* in the binary search and perform a logistic regression on these data points. We report the value of *k* that first reaches 90% reconstruction probability predicted by the logistic regression. If no *k* is sufficient up to the max value, then we deem that the necessary value of *k* is too large for our simulations to discern and we report a missing value. To calculate the point-wise confidence intervals used in Figs. 2, 3, and *SI Appendix*, Fig. S3 for each regression on a set of parameters, we calculate the upper and lower bounds of the 95% CI from the regression coefficients using the delta method. Then, we take the upper bound on the necessary *k* as the first *k* where the lower bound exceeds 90%, and we take the lower bound as the first *k* where the upper bound exceeds 90%.

A validation of the values of *k* found via this method via simulations with more trees and lineage-tracing datasets is provided in *SI Appendix*, Fig. S2 and section A.3.2.

## Supplementary Material

Appendix 01 (PDF)

## Data Availability

Results and algorithms were generated by the authors. Simulated results and code data have been deposited in Github (https://github.com/YosefLab/theoretical_lineage_tracing_reproducibility).
