# Supplementary material for: Theoretical guarantees for phylogeny inference from single-cell lineage tracing
Source: Proc Natl Acad Sci U S A. 2023 Mar 16;120(12):e2203352120. doi: 10.1073/pnas.2203352120 (PMC10041172; doi:10.1073/pnas.2203352120)
Supplement: Supplementary file 1 — Appendix 01 (PDF) [file pnas.2203352120.sapp.pdf]

A Supplemental Information Appendix

A.1 Proofs

A.1.1 Analysis of the δ Function

Since $\delta(d)$ is the only part of the bound that varies according to depth, any universal threshold to the oracle decision must take it into account. It can be verified that $\frac{d^2}{dx^2}\delta(x) \geq 0$ which means δ is convex. Setting $\delta'(x) = 0$, we have that the minimum of δ occurs at:

$$0 = 0.6(-\lambda e^{-\lambda x}(1-q) + q\lambda e^{\lambda x - 2\lambda})$$
$$e^{-2\lambda x} = \frac{q}{(1-q)e^{2\lambda}}$$
$$x = \frac{1}{2\lambda}\ln(\frac{1-q}{q}) + 1$$

Let x^* be the minimum of δ. If $q < 1/2$, then $\ln(\frac{1-q}{q}) > 0$, which means that $x^* > 1$, so on the interval $[0, 1]$, the minimum occurs at $x = 1$, at which point $\delta(x) = 0.6 \cdot e^{-\lambda}$. On the other hand, if $q \geq 1/2$, then the minimum will occur at $\delta(x^*) = 0.6 \cdot 2e^{-\lambda}\sqrt{q(1-q)}$ if $x^* \in [0, 1]$ and $\delta(0) = 0.6 \cdot (1 - q + qe^{-2\lambda})$ if $x^* < 0$. Note, x^* gets smaller as $q \to 1$, so if $q = 1$, then the minimum is precisely $0.6 \cdot e^{-2\lambda}$. Let $d^* \in [0, 1]$ be an arbitrary depth, and let $\delta^*(d^*, q, \lambda) = \min_{x\in[0,d^*]} \delta(x)$. We then have:

$$\delta^* = 0.6 \cdot \begin{cases} (1-q) + qe^{-2\lambda} & \text{if } \frac{1}{2\lambda}\ln(\frac{1-q}{q}) + 1 < 0 \\ 2e^{-\lambda}\sqrt{q(1-q)} & \text{if } \frac{1}{2\lambda}\ln(\frac{1-q}{q}) + 1 \in [0, d^*] \\ e^{-\lambda d^*}(1-q) + qe^{-\lambda(2-d^*)} & \text{if } \frac{1}{2\lambda}\ln(\frac{1-q}{q}) + 1 > d^* \end{cases}$$

A.1.2 Proof of lemma 1:

Let $(a, b|c)$ be a triplet with $depth(LCA(a, b, c)) = d$, and let $\alpha = dist(LCA(a, b, c), LCA(a, b))$. Let $Y = s(a, b)$ and $X = s(b, c)$. For a particular character χ_i, let $Y_i = \mathbb{1}_{\chi_i(a)=\chi_i(b)}$ and let $X_i = \mathbb{1}_{\chi_i(b)=\chi_i(c)}$. We use the following results:

lemma 4 *If for every character χ_i, $P[Y_i - X_i = -1]$ is a decreasing function of α and $P[Y_i - X_i = 1]$ is an increasing function of α for all $\alpha \in [0, 1]$, then for any t, $P[Y - X \geq t]$ is an increasing function of α.*

Proof: For a given character χ_i, $Y_i - X_i = \mathbb{1}_{\chi_i(a)=\chi_i(b)} - \mathbb{1}_{\chi_i(b)=\chi_i(c)}$ has 3 possible outcomes: $\{1, 0, -1\}$. Thus, if $P[Y_i - X_i = -1]$ is a decreasing function of α and $P[Y_i - X_i = 1]$ is an increasing function of α for all $\alpha \in [0, 1]$, then $P[Y_i - X_i \geq t]$ for any t is an increasing function of α. Stating that $P[Y_i - X_i \geq t]$ is an increasing function of α is identical to stating that for any α_1 and α_2 such that $\alpha_1 \geq \alpha_2$, $P_{\alpha_1}[Y_i - X_i \geq t] \geq P_{\alpha_2}[Y_i - X_i \geq t]$. We use the known result from probability theory that for random variables $A_i \overset{iid}{\sim} A$ and $B_i \overset{iid}{\sim} B$ such $P[A \geq t] \geq P[B \geq t]$ for all t, then $P[\sum_i A_i \geq t] \geq P[\sum_i B_i \geq t]$. Thus, as $Y - X = \sum_i Y_i - X_i$ and $Y_i - X_i$ is independent and identically distributed as we assume each character operates independently and identically, then $P_{\alpha_1}[Y - X \geq t] \geq P_{\alpha_2}[Y - X \geq t]$. Thus, $P[Y - X \geq t]$ is an increasing function of α for any t.

lemma 5 *For every character χ_i, $P[Y_i - X_i = -1]$ is a decreasing function of α and $P[Y_i - X_i = 1]$ is an increasing function of α for all $\alpha \in [0, 1]$. Additionally, this result holds in both the general case and stochastic-only missing data cases.*

Proof: We prove this lemma in each case:

Case with no missing data: First, we examine $P[Y_i - X_i = -1]$. $Y_i - X_i = -1$ for a character χ_i corresponds to that character acquiring the same mutation on both the path from $LCA(a, b)$ to b and the path from $LCA(a, b, c)$ to c, and not acquiring that mutation on the path from $LCA(a, b)$ to a. Additionally,

no mutation must be acquired at χ_i on the path from the r to $LCA(a,b,c)$ nor the path from $LCA(a,b,c)$ to $LCA(a,b)$. Thus we have:

$$P[Y_i - X_i = -1] = \sum_j e^{-\lambda d} e^{-\lambda \alpha}(1 - e^{-\lambda(1-d-\alpha)})q_j(1 - e^{-\lambda(1-d)})q_j(1 - (1 - e^{-\lambda(1-d-\alpha)})q_j)$$

Taking only the terms that depend on α, we have:

$$e^{-\lambda\alpha}(1 - e^{-\lambda(1-d-\alpha)})q_j(1 - (1 - e^{-\lambda(1-d-\alpha)})q_j)$$

To show that this value decreases with α, we show that the first derivative is negative with respect to α. We use the following form of the derivative:

$$\lambda(q_j - 1)q_j e^{2\lambda - \lambda(\alpha+2)} - \lambda q_j^2 e^{2\lambda(d+\alpha)-\lambda(\alpha+2)}$$

This value is positive owing to the fact that $q_j \in [0,1]$ for all j. Thus, the function within the summation is decreasing in terms of α. Using the fact that the summation of decreasing functions is decreasing, the overall function is thus decreasing in terms of α.

Secondly, we examine $P[Y_i - X_i = 1]$. $Y_i - X_i = 1$ for χ_i corresponds to a mutation occurring in both a and b, but not in c. A mutation can occur in a and b if it appears on the path from $LCA(a,b,c)$ to $LCA(b,c)$, or if it appears independently in the paths from $LCA(a,b)$ to both a and b. Additionally, this mutation cannot appear on the path from $LCA(a,b,c)$ to c, and no mutations can occur on the path from r to $LCA(a,b,c)$. Thus, we have:

$$P[Y_i - X_i = 1] = \sum_j e^{-\lambda d}((1 - e^{-\lambda\alpha})q_j + e^{-\lambda\alpha}((1 - e^{-\lambda(1-d-\alpha)})q_j)^2)(1 - (1 - e^{-\lambda(1-d)})q_j)$$

Taking on the terms that depend on α, we have:

$$(1 - e^{-\lambda\alpha}) + e^{-\lambda\alpha}(1 - e^{-\lambda(1-d-\alpha)})^2 q_j$$

To show that this value is increasing with α, we show that the first derivative is positive with respect to α. We use the following form of the derivative:

$$\lambda q e^{2\lambda(d+\alpha)-\lambda(\alpha+2)} - \lambda(q-1)e^{2\lambda-\lambda(\alpha+2)}$$

This value is positive owing to the fact that $q_j \in [0,1]$. Thus, the function within the summation is decreasing in terms of α. Using the fact that the summation of decreasing functions is decreasing, the overall function is thus decreasing in terms of α.

General Missing Data Case: Next, we examine the general case with both stochastic and heritable missing data. In this case, we define $s(a,b)$ (analogously $s(b,c)$) as the number of characters shared by a, b that do not have dropout in either a, b or c. As we now simply condition on the fact that a, b, c must all be present, we add an additional $(1 - p_d)$ term to both $P[Y_i - X_i = 1]$ and $P[Y_i - X_i = -1]$. As this term does not depend on α, both functions depend on α as they do in the case without missing data.

Stochastic-only Missing Data Case: Finally, we examine the case with only stochastic missing data. Here we define $s(a,b)$ as the number of mutations shared by a and b in characters that did not suffer dropout in either sample. Thus, in analyzing $Y_i - X_i$ we must consider additional cases in which dropout in one cell can hide the fact that two cells inherited the same mutation.

First, we examine $P[Y_i - X_i = -1]$. $Y_i - X_i = -1$ for a character χ_i corresponds to that character acquiring the same mutation in b and c, not acquiring dropout in neither b nor c, and either observing dropout or not observing that mutation in a. For this to occur, a mutation can occur on the path from r to $LCA(a,b,c)$ while a acquires dropout, or the same mutation can occur on the path from $LCA(a,b,c)$ to $LCA(a,b)$ and the path from $LCA(a,b,c)$ to c while a acquires dropout, or the mutation can occur on the

path from $LCA(a, b)$ to b and the path from $LCA(a, b, c)$ to c while not appearing in a. The probability of this is:

$$\sum_j (1-p_d)^2 (p_d(1-e^{\lambda d})+e^{\lambda d}(1-e^{-\lambda(1-d)})q_j(p_d(1-e^{-\lambda\alpha})q_j+e^{-\lambda\alpha}(1-e^{-\lambda(1-d-\alpha)})q_j(1-(1-p_d)(1-e^{-\lambda(1-d-\alpha)}))q_j)$$

Taking the terms that depend on α:

$$p_d(1-e^{-\lambda\alpha})q_j + e^{-\lambda\alpha}(1-e^{-\lambda(1-d-\alpha)})q_j(1-(1-p_d)(1-e^{-\lambda(1-d-\alpha)}))q_j)$$

To show that this value decreases with α, we show that the first derivative is negative with respect to α. We use the following form of the derivative:

$$\lambda q_j(p_d - 1)e^{-\lambda(\alpha+2)}(q_j e^{2m(d+\alpha)} - e^{2\alpha}(q_j - 1))$$

This value is positive owing to the fact that $q_j \in [0, 1]$ for all j and that $p_d \in [0, 1)$. Thus, the function within the summation is decreasing in terms of α. Using the fact that the summation of decreasing functions is decreasing, the overall function is thus decreasing in terms of α.

Secondly, we examine $P[Y_i - X_i = 1]$. $Y_i - X_i = 1$ for χ_i corresponds to a mutation occurring in both a and b, not acquiring dropout in neither a nor b, and either observing dropout or not observing that mutation in c. For this to occur, a mutation can occur on the path from r to $LCA(a, b, c)$ while c acquires dropout, on the path from $LCA(a, b, c)$ to $LCA(a, b)$ while the mutation is not acquired in c or c acquires dropout, or the mutation occurs independently on the path from $LCA(a, b)$ to a and b while not appearing in c. The probability of this is:

$$\sum_j (1-p_d)^2(p_d(1-e^{-\lambda d})q_j + e^{-\lambda d}(1-(1-p_d)(1-e^{-\lambda(1-d)}))q_j((1-e^{-\lambda\alpha})q_j + e^{-\lambda\alpha}((1-e^{-\lambda(1-\alpha-d)})q_j)^2))$$

Taking on the terms that depend on α, we have:

$$(1-e^{-\lambda\alpha}) + e^{-\lambda\alpha}(1-e^{-\lambda(1-d-\alpha)})^2 q_j$$

Note that this is the same value as above in the case without missing data, and hence the function will have the same dependence on α as in that case. Hence, the function is overall decreasing with α.

Proof: By lemmas 4 and 5, $P[s(a, b) - s(a, c) \geq t]$ is an increasing function of α. Thus, the minimum value of α results in the minimum value of $P[s(a, b) - s(a, c) \geq t]$. This value occurs at $\alpha = \ell^*$, showing lemma 1.

A.1.3 Proof of lemma 2:

First, we will show that condition $i)$ will hold with probability $1 - \zeta$ if:

$$\frac{ke^{\lambda d}\lambda(\ell^*\delta(d))^2}{32(\ell^* + (1-e^{-\lambda})q)} \geq 3\log(n) + \log 1/\zeta$$

To see this, first note that $dist(LCA(a, b, c), LCA(b, c)) \geq \ell^*$. By lemma 1, we can WLOG assume that $dist(LCA(a, b, c), LCA(b, c)) = \ell^*$ because that is the worst case, i.e. the case where $P[s(a, b) - s(b, c) \geq t]$ is minimized. Any condition sufficient for this case will be sufficient overall. Let $Y = s_c(a, b)$ and $X = s_a(b, c)$. Since $E(Y) - E(X) \geq k\lambda\ell^*\delta(d) \geq k\lambda\ell^*\delta^* = 2t$, in order to ensure that $Y - X \geq t$, it suffices to have $Y - X \geq k\lambda\ell^*\delta(d)/2$ which holds when $Y \geq E(Y) - k\lambda\ell^*\delta(d)/4$ and $X \leq E(X) + k\lambda\ell^*\delta(d)/4$. Thus, we have

$$P[Y - X < t] \leq P[Y < E(Y) - k\lambda\ell^*\delta(d)/4] + P[X > E(X) + k\lambda\ell^*\delta(d)/4]$$

To bound the probability of both events using the above versions of Hoeffding's inequality, we have:

$$\begin{aligned}
P[Y < E(Y) - k\lambda\ell^*\delta(d)/4] &= Pr[Y \leq E(Y)(1 - \frac{k\lambda\ell^*\delta(d)}{4E(Y)})] \\
&\leq exp(-\frac{k^2\lambda^2\ell^{*2}\delta(d)^2}{32E(Y)}) \\
&\leq exp(-\frac{k^2\lambda^2\ell^{*2}\delta(d)^2}{32ke^{-\lambda d}(\lambda\ell^* + \lambda(1-e^{-\lambda})q)}) \\
P[X > E(X) + k\lambda\ell^*\delta(d)/4] &= Pr[X \geq E(X)(1 + \frac{k\lambda\ell^*\delta(d)}{4E(X)})] \\
&\leq exp(-\frac{k^2\lambda^2\ell^{*2}\delta(d)^2}{32E(X) + 4k\lambda\ell^*\delta(d)}) \\
&\leq exp(-\frac{e^{\lambda d}k^2\lambda^2\ell^{*2}\delta(d)^2}{32k(\lambda\ell^* + \lambda(1-e^{-\lambda})q)})
\end{aligned}$$

The last line follows from the fact that $\delta(d) \leq e^{-\lambda d}$ and $E(X) \leq ke^{-\lambda d}(1-e^{-\lambda})^2 q \leq ke^{-\lambda d}\lambda(1-e^{-\lambda})q$. Since $e^{\lambda d}\delta(d) = 0.6(1 - q + qe^{-2\lambda(1-d)}) \geq 0.6(1 - q + qe^{-2\lambda})$, in order to ensure that both bad events have probability at most ζn^{-3}, it suffices to take

$$\frac{0.6k\lambda(1-q+qe^{-2\lambda})\delta(d)\ell^{*2}}{32(\ell^* + (1-e^{-\lambda})q)} \geq 3\log(n) + \log 1/\zeta$$

Since $\delta^* \leq \delta(d)$, the above condition holds as long as

$$k \geq \frac{(96\log n + 32\log 1/\zeta)(\ell^* + (1-e^{-\lambda})q)}{0.6\lambda\ell^{*2}\delta^*(1-q+qe^{-2\lambda})}$$

Applying the same argument to $s_c(a,b) - s_b(a,c)$ and combining both results gives $P[s_c(a,b) - \max(s_b(a,c), s_a(b,c)) < t] \leq 4\zeta n^{-3}$. Taking a union bound over all $\binom{n}{3} = O(n^3)$ triplets, we see that the probability of condition $i)$ failing for any triplet is at most ζ.

To get guarantees on the second condition, note that condition $i)$ implies that condition $ii)$ holds for all triplets separated by an edge of length at least ℓ^*. Thus we can focus on the triplets that are not covered by condition $i)$. Let $(a,b|c)$ be an arbitrary triplet such that $dist(LCA(a,b,c), LCA(a,b)) < \ell^*$ and again let $Y = s_c(a,b)$, $X = s_a(b,c)$ and d be the depth of $LCA(a,b,c)$ (note that we are focusing WLOG on $s_a(b,c)$ since it has the same distribution as $s_b(a,c)$). We want to show that with high probability, $X - Y < t$. Again, it suffices to upper bound $P[Y \leq E(Y) - t/2]$ and $P[X \geq E(X) \geq t/2]$ because $E(Y) \geq E(X)$. Note that we have already bounded the second quantity. To bound the first quantity, note that the worst case scenario is that $dist(LCA(a,b,c), LCA(a,b))$ is as small as possible. Since in this lemma we make no assumption on the minimal edge length, this quantity can be arbitrarily small and in the worst case, $dist(LCA(a,b,c), LCA(a,b)) = 0$, which means Y has the same distribution as X. Note that this case technically cannot happen as it would imply that $\mathcal{T}$ has a trifurcating branch but it is possible to get arbitrarily close to this case with no restrictions on edge lengths. This gives:

$$\begin{aligned}
Pr[Y \leq E(Y) - k\lambda\ell^*\delta^*/4] &\leq Pr[X \leq E(X) - k\lambda\ell^*\delta^*/4] \\
&= Pr[X \leq E(X)(1 - \frac{k\lambda\ell^*\delta^*}{4E(X)})] \\
&\leq exp(-\frac{k^2\lambda^2\ell^{*2}\delta^{*2}}{32ke^{-\lambda d}\lambda^2(1-d)^2 q}) \\
&\leq exp(-\frac{k\ell^{*2}\delta^{*2}}{32q})
\end{aligned}$$

Thus, if we take:

$$k \geq \max\Big(\frac{(96\log n + 32\log 1/\zeta)q}{\ell^{*2}\delta^{*2}}, \frac{(96\log n + 32\log 1/\zeta)(\ell^* + (1-e^{-\lambda})q)}{0.6\lambda\ell^{*2}\delta^*(1-q+qe^{-2\lambda})}\Big)$$

then we have $P[X - Y \geq t] < \zeta n^{-3}$. By symmetry, this means $P[s_a(b,c) - s_c(a,b) \geq t \bigcup s_b(a,c) - s_c(a,b) \geq t] \leq 2\zeta n^{-3}$ Since we can union bound over one bad event of probability at most $2\zeta n^{-3}$ for each of the $\binom{n}{3}$ triplets, we have that conditions $i)$ and $ii)$ both hold with probability at least $1 - \zeta$.

A.1.4 Proof of lemma 3:

Given a triplet $(a, b|c)$, with LCA at depth at most d^*, we defined ϵ to be the probability that no dropout occurs in a particular character of all three cells. First we will prove that $\epsilon \geq (1 - p_d)^3$, which is to say the dropout events are positively correlated. Let p_h be the probability that heritable dropout occurs on the path from r to a. Let p_b be the probability that a heritable dropout occurs on the path from $LCA(a, b)$ to b given that no dropout has occurred on the path from r to $LCA(a, b)$ and define p_c similarly. Note that $p_b \leq p_c \leq p_h$ since the probability a dropout occurs along a path increases with the length of the path. Let p_s be the probability that a stochastic dropout occurs at given character on a leaf given that no heritable dropout has occurred yet on that character. Then we have $\epsilon = (1-p_h)(1-p_b)(1-p_a)(1-p_s)^3 \geq ((1-p_h)(1-p_s))^3 = (1-p_d)^3$. Since at least one of the cells in the triplet needs to not incur dropout at a character in order for all three of them to have no dropout, we also have $\epsilon \leq 1 - p_d$.

To prove the bounds on k we will proceed as in the proof of lemma 2 and assume that $dist(LCA(a, b, c), LCA(a, b)) = \ell^*$, noting that lemma 5 extends the result of lemma 1 to the general case with missing data. Let $Y = s_c(a, b)$ and $X = s_a(b, c)$. Thus, we have:

$$\begin{aligned}
Pr[Y \leq E(Y) - k\epsilon\lambda\ell^*\delta(d)/4] &= Pr[Y \leq E(Y)(1 - \frac{k\epsilon\lambda\ell^*\delta(d)}{4E(Y)})] \\
&\leq exp(-\frac{\epsilon^2 k^2\lambda^2\ell^{*2}\delta(d)^2}{32E(Y)}) \\
Pr[X \geq E(X) + k\epsilon\lambda\ell^*\delta(d)/4] &= Pr[X \geq E(X)(1 + \frac{k\epsilon\lambda\ell^*\delta(d)}{4E(X)})] \\
&\leq exp(-\frac{\epsilon^2 k^2\lambda^2\ell^{*2}\delta(d)^2}{32E(X) + 4k\lambda\ell^*\delta(d)})
\end{aligned}$$

Since $E(Y) \leq k\epsilon e^{-\lambda d}\lambda\ell^*$ and $E(X) \leq k\epsilon e^{-\lambda d}\lambda^2 q$, we see that both probabilities are at most $n^{-3}\zeta$ when

$$\frac{0.6k(1-p_d)^3\lambda(1-q+qe^{-2\lambda})\delta(d)\ell^{*2}}{32(\ell^* + (1-e^{-\lambda})q)} \geq \frac{0.6k\epsilon\lambda(1-q+qe^{-2\lambda})\delta(d)\ell^{*2}}{32(\ell^* + (1-e^{-\lambda})q)} \geq 3\log(n) + \log 1/\zeta$$

If both bad events don't happen, then we have $Y - X \geq k\epsilon\lambda\ell^*\delta^*/2 \geq k(1-p_d)^3\lambda\ell^*\delta^*/2 = t$. This gives the necessary bound for condition $i)$ to hold.

To get guarantees on the second condition, let $X = s_b(a, c)$ for any triplet a, b, c whose LCA has depth at most d^* and where c is the outgroup. we have that

$$\begin{aligned}
Pr[X \leq E(X) - k(1-p_d)^3\lambda\ell^*\delta^*/4] &= Pr[X \leq E(X)(1 - \frac{k(1-p_d)^3\lambda\ell^*\delta^*}{4E(X)})] \\
&\leq exp(-\frac{k(1-p_d)^6\ell^{*2}\delta^{*2}}{32\epsilon q}) \\
&\leq exp(-\frac{k(1-p_d)^5\ell^{*2}\delta^{*2}}{32q})
\end{aligned}$$

To ensure that this probability is at most $n^{-3}\zeta$, it suffices to take

$$k \geq \frac{(96\log n + 32\log 1/\zeta)q}{\ell^{*2}\delta^{*2}(1-p_d)^5}$$

The rest of the argument is exactly the same as in the proof of lemma 2.

A.1.5 Proof of Theorem 3

To prove Theorem 3, we must first bound the effects of incorrectly inferred mutations at internal nodes. Assume that r' is an internal node generated in line 5 of the algorithm. Further assume that the tree rooted by r' is correct (i.e., it is a sub-graph of $\mathcal{T}$). The process of assigning a mutation state to r' (lines 6–12) may incorrectly include mutations that emerged independently at its two child trees. To account for this, we define $P_{i,j}(r')$ to be the probability that every leaf beneath r' has character i mutated to state j, given that character i is not mutated at r'. This probability is bounded according to the following lemma (proof shown later).

lemma 6 *Given the constraints on λ, ℓ and q in Theorem 3, it follows that $P_{i,j}(r') \leq 2\lambda^2 c\ell q_j^2$ for any internal node $r' \in \mathcal{T}$, character i and state j.*

To guarantee that the algorithm has a low probability of making mistakes in forming cherries (line 13), we focus on a pair of "active" nodes u, w in a given iteration of the algorithm (i.e., nodes that are roots of trees in the current set S; defined in lines 2 and 14). We assume that up until this point, the algorithm did not form any incorrect cherries (i.e., all trees in S are sub-graphs of $\mathcal{T}$). It suffices to show that with high probability, if u and w do not form a cherry in $\mathcal{T}$, there must be some internal node, v on the path from $r' = LCA(u, w)$ to u such that $s(u, v) > s(u, w)$ (note that r' is LCA in $\mathcal{T}$). If this holds, then if u, w are the first incorrect pair to be merged, then there must be some descendent u' of v such that $s(u, u') \geq s(u, v) > s(u, w)$, contradicting the fact that u, w was the most similar pair. Note that u and w can be either leaves or internal nodes.

Let d be the depth of r' and let $\alpha = dist(r', v)$. Since the maximum edge length is $\sqrt{c\ell}$, we have WLOG that $dist(r', u), dist(r', w) \leq \alpha + \sqrt{c\ell}$ (if $dist(r', w)$ is greater than this quantity we can simply switch the roles of u and w and redefined α to be the distance from r' to the parent of w). Let Z be the number of mutations that occurred on the path from r' to v. This gives:

$$E(Z) = ke^{-\lambda d}(1 - e^{-\lambda\alpha})$$

Note that due to irreversibility the mutations tallied in Z will be present in all nodes under v, including u and u'. Let X be the number of character assignments that are shared between u and w (assigned by the algorithm per lines 7–11) and that are not present in r' (per the ground truth). There are two ways in which a character can be assigned state j at u (or w). Firstly, in the ground truth tree, a character can mutate to state j on the paths from r' to u (or w), with probability at most $(1 - e^{-\lambda(\alpha+\sqrt{c\ell})})q_j$. In that case, the mutation will be present in all downstream leaves, and thus assigned by the algorithm to u (or to w). Secondly, if it did not mutate on that path, it could instead have mutated in enough places in the sub-tree rooted beneath u (or w) such that every leaf in that sub-tree has the mutation at that character. By lemma 6, we see that the probability of this occurring is at most $2\lambda^2 c\ell q_j^2$. Requiring that in both u and w we have an occurrence of at least one of these scenarios for a given state j and summing over all states, we get:

$$\begin{aligned}
E(X) &\leq ke^{-\lambda d}\sum_j((1 - e^{-\lambda(\alpha+\sqrt{c\ell})})q_j + e^{-\lambda(\alpha+\sqrt{c\ell})}2\lambda^2 c\ell q_j^2)^2 \\
&\leq ke^{-\lambda d}\sum_j((1 - e^{-\lambda(\alpha+\sqrt{c\ell})})q_j + 2\lambda^2 c\ell q_j^2)^2 \\
&= ke^{-\lambda d}\sum_j(1 - e^{-\lambda(\alpha+\sqrt{c\ell})} + 2\lambda^2 c\ell q_j)^2 q_j^2 \\
&\leq ke^{-\lambda d}(1 - e^{-\lambda(\alpha+\sqrt{c\ell})} + 2\lambda^2 c\ell \max_j q_j)^2 q
\end{aligned}$$

Let $q_{\max} = \max_j q_j$. Assume $E(Z) > E(X)$ and let $\Delta = E(Z) - E(X)$. Again by the above versions of Hoeffding's inequality, we have the following concentration inequalities:

$$
\begin{aligned}
Pr[Z < E[Z] - \Delta/2] &\leq exp(-\frac{\Delta^2}{8E(Z)}) \\
&\leq exp(-\frac{\Delta}{10E(Z)}) \\
Pr[X \geq E(X) + \Delta/2] &\leq exp(-\frac{\Delta^2}{8E(X) + 2\Delta}) \\
&\leq exp(-\frac{\Delta^2}{8E(X) + 2(E(Z) - E(X))}) \\
&\leq exp(-\frac{\Delta}{10E(Z)})
\end{aligned}
$$

Suppose $\lambda q \leq \frac{\beta}{\max(1,c)}$. Let $\gamma = 2\sqrt{c\ell}$. Then, we have:

$$
\begin{aligned}
\Delta &\geq ke^{-\lambda d}(1 - e^{-\lambda\alpha} - (1 - e^{-\lambda(\alpha+\sqrt{c\ell})} + 2\lambda^2 c\ell q_{\max})^2 q) \\
&= ke^{-\lambda d}(1 - e^{-\lambda\alpha} - (1 - e^{-\lambda\alpha}e^{-\lambda\sqrt{c\ell}} + 2\lambda^2 c\ell q_{\max})^2 q) \\
&\geq ke^{-\lambda d}(1 - e^{-\lambda\alpha} - (1 - e^{-\lambda\alpha}(1 - \lambda\sqrt{c\ell}) + 2\lambda^2 c\ell q_{\max})^2 q) \\
&= ke^{-\lambda d}(1 - e^{-\lambda\alpha} - (1 - e^{-\lambda\alpha} + e^{-\lambda\alpha}\lambda\sqrt{c\ell} + 2\lambda^2 c\ell q_{\max})^2 q) \\
&= ke^{-\lambda d}(1 - e^{-\lambda\alpha} - (1 - e^{-\lambda\alpha} + \lambda\sqrt{c\ell}(e^{-\lambda\alpha} + \lambda\gamma q_{\max}))^2 q) \\
&= ke^{-\lambda d}(1 - e^{-\lambda\alpha} - (1 - e^{-\lambda\alpha})^2 q - 2\lambda(1 - e^{-\lambda\alpha})(e^{-\lambda\alpha} + \lambda\gamma q_{\max})\sqrt{c\ell}q - \lambda^2(e^{-\lambda\alpha} + \lambda\gamma q_{\max})^2 c\ell q) \\
&\geq ke^{-\lambda d}((1 - e^{-\lambda\alpha})(1 - (1 - e^{-\lambda\alpha})q - \beta\gamma(e^{-\lambda\alpha} + \lambda\gamma q_{\max})) - \beta\lambda(e^{-\lambda\alpha} + \lambda\gamma q_{\max})^2\ell) \\
&\geq ke^{-\lambda d}((1 - e^{-\lambda\alpha})(1 - \lambda\alpha q - \beta\gamma(e^{-\lambda\alpha} + \lambda\gamma q_{\max})) - \beta\lambda(e^{-\lambda\alpha} + \lambda\gamma q_{\max})^2\ell) \\
&\geq ke^{-\lambda d}((1 - e^{-\lambda\alpha})(1 - \beta(\alpha + \gamma e^{-\lambda\alpha} + \lambda\gamma^2 q_{\max})) - \beta\lambda(e^{-\lambda\alpha} + \lambda\gamma q_{\max})^2\ell) \\
&\geq ke^{-\lambda d}((1 - e^{-\lambda\alpha})(1 - \beta(1 + \gamma e^{-\lambda} + \lambda\gamma^2 q_{\max})) - \beta\lambda(e^{-\lambda\alpha} + \lambda\gamma q_{\max})^2\ell)
\end{aligned}
$$

Where the last line follows from the fact that the maximum of the function $\alpha + \gamma e^{-\lambda\alpha}$ occurs at $\alpha = 1$.

Now, it remains to find a bound on β so that $\Delta > 0$ and $\frac{\Delta^2}{E[Z]}$ is lower bounded. Let $C = \gamma e^{-\lambda} + \lambda\gamma^2 q_{\max}$. To ensure that $\Delta > 0$, we see that the last line from the previous block needs to be > 0. Taking this inequality and rearranging terms, it suffices to show that:

$$
1 > \beta(1 + C + \frac{\lambda\ell(e^{-\lambda\alpha} + \lambda\gamma q_{\max})^2}{1 - e^{-\lambda\alpha}})
$$

Note that

$$
\frac{\lambda\ell(e^{-\lambda\alpha} + \lambda\gamma q_{\max})^2}{1 - e^{-\lambda\alpha}} \leq \frac{\lambda\ell(e^{-\lambda\ell} + \lambda\gamma q_{\max})^2}{1 - e^{-\lambda\ell}} \approx (e^{-\lambda\ell} + \lambda\gamma q_{\max})^2
$$

so our sufficient condition can be written as

$$
1 > \beta(1 + C + (e^{-\lambda\ell} + \lambda\gamma q_{\max})^2)
$$

We lower bound $\frac{\Delta^2}{E[Z]}$ by the following:

$$\frac{\Delta^2}{E[Z]} \geq \min_{\alpha\in[\ell,1]} \frac{k^2e^{-2\lambda d}((1-e^{-\lambda\alpha})(1-\beta(1+C)) - \beta\lambda(e^{-\lambda\alpha}+\lambda\gamma q_{\max})^2\ell)^2}{ke^{-\lambda d}(1-e^{-\lambda\alpha})}$$
$$= \frac{k^2e^{-2\lambda d}((1-e^{-\lambda\ell})(1-\beta(1+C)) - \beta\lambda(e^{-\lambda\ell}+\lambda\gamma q_{\max})^2\ell)^2}{ke^{-\lambda d}(1-e^{-\lambda\ell})}$$
$$\geq ke^{-\lambda d}\left(\frac{(1-e^{-\lambda\ell})^2(1-\beta(1+C))^2 - 2(1-e^{-\lambda\ell})(1-\beta(1+C))\beta\lambda\ell(e^{-\lambda\ell}+\lambda\gamma q_{\max})^2}{(1-e^{-\lambda\ell})} + \frac{(\beta\lambda\ell(e^{-\lambda\ell}+\lambda\gamma q_{\max})^2)^2}{(1-e^{-\lambda\ell})}\right)$$
$$\geq ke^{-\lambda d}((1-e^{-\lambda\ell})(1-\beta(1+C))^2 - 2(1-\beta(1+C))\beta\lambda\ell(e^{-\lambda\ell}+\lambda\gamma q_{\max})^2)$$
$$\approx ke^{-\lambda d}\lambda\ell((1-\beta(1+C))^2 - 2(1-\beta(1+C))\beta(e^{-\lambda\ell}+\lambda\gamma q_{\max})^2)$$
$$= ke^{-\lambda d}\lambda\ell(1-\beta(1+C))(1-\beta(1+C) - 2\beta(e^{-\lambda\ell}+\lambda\gamma q_{\max})^2)$$

In order for this bound to be positive, we need

$$1 > \beta(1+C+2(e^{-\lambda\ell}+\lambda\gamma q_{\max})^2)$$

Note that this condition satisfies the above condition on β and thus would imply $E[Z] > E[X]$. Thus, if

$$\beta < \frac{1}{1+C+2(e^{-\lambda\ell}+\lambda\gamma q_{\max})^2} = \frac{1}{1+2\sqrt{c\ell}e^{-\lambda}+4\lambda c\ell q_{\max}+2(e^{-\lambda\ell}+2\lambda\sqrt{c\ell}q_{\max})^2}$$

both Δ and $\frac{\Delta^2}{E[Z]}$ are strictly positive.

To bound the probability that $Z < E[Z] - \Delta/2$ and $X \geq E(X) + \Delta/2)$ is at most $n^{-2}\zeta$, we need:

$$exp(-\frac{\Delta^2}{10E[Z]}) \leq n^{-2}\zeta$$

This is satisfied if k satisfies the following:

$$k \geq \frac{20\log n + 10\log(1/\zeta)}{\lambda e^{-\lambda}\ell(1-\beta(1+C))(1-\beta(1+C)-2\beta(e^{-\lambda\ell}+\lambda\gamma q_{\max})^2)}$$

In other words, for any pair of vertices u, w that are not children of the same node, there will be a vertex u' that such that $LCA(u,u')$ is a descendent of $LCA(u,w)$ and $P[s(u,u') \leq s(u,w)] \leq 2n^{-2}\zeta$. If $s(u,u') > s(u,w)$, then (u,w) cannot be the first pair of incorrectly joined vertices. Taking a union bound over at most $n^2/2$ pairs of vertices, we see that with with probability at least $1-\zeta$, there is no first pair of incorrectly joined vertices, which means the algorithm is correct.

A.1.6 Proof of lemma 6:

For this proof, we use the following results:

lemma 7 *Let ρ be the maximum edge length in $\mathcal{T}$. For any character, state pair (i,j), if there exists a number $p > 0$ which satisfies $((1-e^{-\lambda\rho})q_j + p)^2 \leq p$, then p is an upper bound on $P_{i,j}(v)$ for any node $v \in \mathcal{T}$.*

Proof: We will proceed by induction on $\mathcal{T}$. Suppose v is a leaf. Then $P_{i,j}(v) = 0$, since if the ith character does not mutate, it cannot take on state j. Now let v be an arbitrary non-leaf vertex, with children u and w. By our inductive hypothesis, $P_{i,j}(w) \leq p$ and $P_{i,j}(u) \leq p$. Since the length of the edge from v to either of it's children is at most ρ, the probability that the character mutates to state j on either edge is at most $(1-e^{-\lambda\rho})q_j$. Thus, if we condition on the fact that χ_i is not mutated on v, we have:

$$\begin{aligned} P_{i,j}(v) &\leq ((1-e^{-\lambda\rho})q_j + e^{-\lambda\rho}P_{i,j}(u))((1-e^{-\lambda\rho})q_j + e^{-\lambda\rho}P_{i,j}(w)) \\ &\leq ((1-e^{-\lambda\rho})q_j + P_{i,j}(u))((1-e^{-\lambda\rho})q_j + P_{i,j}(w)) \\ &\leq ((1-e^{-\lambda\rho})q_j + p)^2 \\ &\leq p \end{aligned}$$

Where the last inequality follows from our assumption on p. Given the above lemma, we can simply solve for p to find an upper bound on all $P_{i,j}(v)$ in $\mathcal{T}$.

lemma 8 *Suppose*

$$\rho \leq -\frac{1}{\lambda}\ln[(1-\frac{3}{16\max_j(q_j)})]$$

Then we have $P_{i,j}(v) \leq 2\lambda^2\rho^2 q_j^2$ *for any node* $v \in \mathcal{T}$

Proof: Let $y = (1-e^{-\lambda\rho})q_j$. Note that by our above assumption, $y \leq 3/16$. By our above assumption, we have:

$$\begin{aligned}
\rho &\leq -\frac{1}{\lambda}\ln[(1-\frac{3}{16\max_j(q_j)})] \\
(1-e^{-\lambda\rho})\max_j(q_j) &\leq \frac{3}{16} \\
(1-e^{-\lambda\rho})q_j &\leq \frac{3}{16} \\
y &\leq \frac{3}{16}
\end{aligned}$$

Next, by lemma 7, we know that if there is a $p > 0$ such that $(y+p)^2 \leq p$, then such a p would be an upper bound on all $P_{i,j}(v)$. We can find such a p by setting the inequality to an equality and finding the smallest root of the resulting polynomial.

$$\begin{aligned}
y^2 + 2yp + p^2 &= p \\
y^2 + (2y-1)p + p^2 &= 0
\end{aligned}$$

Our initial assumption of ρ guarantees that $4y < 1$, which means the smallest root of the polynomial above can be given as

$$\begin{aligned}
p &= \frac{1}{2}(1-2y-\sqrt{((2y-1)^2-4y^2)}) \\
&= \frac{1}{2}(1-2y-\sqrt{(4y^2-4y+1-4y^2)}) \\
&= \frac{1}{2}(\sqrt{1-4y+4y^2}-\sqrt{(1-4y)}) \\
&= \frac{1}{2}\frac{4y^2}{\sqrt{1-4y+4y^2}+\sqrt{(1-4y)}}
\end{aligned}$$

Where the last line follows by multiplying the numerator and denominator by $\sqrt{1-4y+4y^2}+\sqrt{(1-4y}$. To upper bound p, we have

$$\begin{aligned}
p &\leq \frac{1}{2}\frac{4y^2}{2\sqrt{1-4y}} \\
&= \frac{y^2}{\sqrt{1-4y}} \\
&\leq 2y^2
\end{aligned}$$

Where the last inequality follows from the fact $y \leq 3/16$, which means the denominator is at least $1/2$. Finally, we have $2y^2 = 2(1-e^{-\lambda\rho})^2 q_j^2 \leq 2\lambda^2\rho^2 q_j^2$.

Now simply take $\rho = \sqrt{c\ell^*}$ and apply lemma 8 to show lemma 6.

A.2 Additional Analyses

A.2.1 Alternative Strategy for Regime with only Stochastic Missing Data

Now we assume that missing data only occurs due to technical difficulties in reading out mutation sequences in cells, i.e. that only stochastic missing data occurs. Specifically, for any given cell and any given Cas9 recording site, we assume that the sequence of this site is missing from our data with probability p_s and that this omission occurs independently from all other cells or mutation sites. With this definition, we consider a slightly modified oracle by defining $s(a, b)$ as the number of mutations shared by a and b in characters that did not suffer dropout in either sample. Note that this definition of $s(a, b)$ is independent of a third cell c unlike in the general case. Accordingly we revise conditions $i)$ and $ii)$ by setting the decision threshold t to be $k(1-p_s)^2\lambda\ell\delta^*$. These modified definitions lead to a more relaxed dependency between the mutation rate and the extent of missing data:

lemma 9 *In the presence of stochastic missing data at a rate of p_s, condition i) holds with probability at least $1-\zeta$ if we we have the following guarantees on the parameters q, λ, ℓ^*, d^*, and k:*

$$k \geq \frac{(96\log n + 32\log 1/\zeta)(\ell^* + (1-e^{-\lambda})q + e^{\lambda d^*}d^*)}{0.6(1-p_s)^2\lambda\ell^{*2}\delta^*(1-q+qe^{-2\lambda})}$$

Both conditions i and ii hold with probability at least $1-\zeta$ if we have:

$$k \geq \max\Big(\frac{(96\log n + 32\log 1/\zeta)(\ell^* + \lambda q + e^{\lambda d^*}d^*)}{0.6(1-p_s)^2\lambda\ell^{*2}\delta^*(1-q+qe^{-2\lambda})}, \frac{(96\log n + 32\log 1/\zeta)(d^* + (1-e^{-\lambda})q)}{(1-p_s)^2\lambda\ell^{*2}\delta^{*2}}\Big)$$

An empirical demonstration of the tightness of lemma 9 using simulations is provided in appendix Figure S4.

Proof: For a triplet $(a, b|c)$ in the case without dropout, any mutation that occurred on the path from the root to $LCA(a, b, c)$ is inherited by each member of the triplet, and thus $s(a, b) - s(b, c) = s_c(a, b) - s_a(b, c)$. But in the case of missing data, this is no longer true as mutations that occurred before $LCA(a, b, c)$ may be obscured by dropout and therefore not present in the character information of a, b, or c. We must now account for these early mutations in our calculations.

Let p_s be the stochastic missing data rate and let $s(a, b)$ be the number of mutations shared between a and b, ignoring characters that have dropout in either a or b. The number of mutations shared by a, b after their divergence is now Binomial(k, p) where p is

$$\geq (1-p_s)^2(1-e^{-\lambda d} + e^{-\lambda d}((1-e^{-\lambda\ell^*}) + e^{-\lambda\ell^*}(1-e^{-\lambda(1-\ell^*-d)})^2q)$$

Here $(1-p_s)^2$ is the probability that this character does not acquire dropout in neither a nor b, $1-e^{-\lambda d}$ is the probability that a given mutation occurred before d, and of the remaining terms the left term is the probability the mutation occurred on the path from $LCA(a, b, c)$ to $LCA(a, b)$, and the right term is the probability a given mutation is shared by a, b due to convergent evolution.

Thus:

$$E[s(a, b)] \geq k(1-p_s)^2(1-e^{-\lambda d} + e^{-\lambda d}((1-e^{-\lambda\ell^*}) + e^{-\lambda\ell^*}(1-e^{-\lambda(1-\ell^*-d)})^2q)$$

Similarly:

$$E[s(b, c)] \leq k(1-p_s)^2(1-e^{-\lambda d} + e^{-\lambda d}(1-e^{-\lambda(1-d)})^2q)$$

Thus, we have that:

$$
\begin{aligned}
E[s(a,b)-s(b,c)] &\geq (1-p_s)^2k(1-e^{-\lambda d}+e^{-\lambda d}((1-e^{-\lambda\ell^*})+e^{-\lambda\ell^*}(1-e^{-\lambda(1-\ell^*-d)})^2q-(1- \\
&e^{-\lambda d}+e^{-\lambda d}(1-e^{-\lambda(1-d)})^2q)) \\
&= (1\text{-p}_s)^2ke^{-\lambda d}(1-e^{-\lambda\ell^*}+q(e^{-\lambda\ell^*}-1+e^{-2\lambda(1-d)+\lambda\ell^*}-e^{-2\lambda(1-d)})) \\
&= (1\text{-p}_s)^2ke^{-\lambda d}((1-e^{-\lambda\ell^*})(1-q)+qe^{-2\lambda(1-d)}(e^{\lambda\ell^*}-1)) \\
&\geq (1-p_s)^2ke^{-\lambda d}((1-e^{-\lambda\ell^*})(1-q)+qe^{-2\lambda(1-d)}\lambda\ell^*) \\
&\geq 0.6(1-p_s)^2k(e^{-\lambda d}\lambda\ell^*(1-q)+qe^{-\lambda(2-d)}\lambda\ell^*) \\
&= 0.6(1\text{-p}_s)^2k\lambda\ell^*(e^{-\lambda d}(1-q)+qe^{-\lambda(2-d)})
\end{aligned}
$$

The fourth line follows from the fact that $0.6x \leq 1-e^{-x} \leq x$ for $x \in [0,1]$. Again taking $\delta(d) = 0.6e^{-\lambda d}(1-q)+qe^{-\lambda(2-d)}$, we then have that for any triplet $(a,b|c)$, where $depth(LCA(a,b,c)) = d$ and $dist(LCA(a,b,c),\ LCA(a,b)) \geq \ell^*$

$$E[s(a,b)-s(b,c)] \geq (1-p_s)^2k\lambda\ell^*\delta(d)$$

First, we will show that condition $i)$ will hold with probability $1-\zeta$ if:

$$\frac{(1-p_s)^2ke^{\lambda d}\lambda(\ell^*\delta(d))^2}{32(\ell^*+(1-e^{-\lambda})q+e^{\lambda d}d)} \geq 3\log(n)+\log 1/\zeta$$

To see this, let $(a,b|c)$ be any triplet at depth at most d^* and the distance between their LCAs be at least ℓ^*. By lemma 1, we can WLOG assume that $dist(LCA(a,b,c),LCA(b,c)) = \ell^*$ because that is the worst case, i.e. the case where $P[s(a,b)-s(b,c)\geq t]$ is minimized. Note that lemma 5 extends the result of lemma 1 to the case with only stochastic missing data. Any condition sufficient for this case will be sufficient overall. Let $Y = s(a,b)$ and $X = s(b,c)$. Since $E(Y)-E(X) \geq (1-p_s)^2k\lambda\ell^*\delta^* = 2t$, in order to ensure that $Y-X \geq t$, it suffices to have $Y > E(Y) = t/2$ and $X < E(X) = t/2$. To show that both occur with high probability, we have:

$$
\begin{aligned}
Pr[Y \leq E(Y)-(1-p_s)^2k\lambda\ell^*\delta(d)/4] &= Pr[Y \leq E(Y)(1-\frac{(1-p_s)^2k\lambda\ell^*\delta(d)}{4E(Y)})] \\
&\leq exp(-\frac{(1-p_s)^4k^2\lambda^2\ell^{*2}\delta(d)^2}{32E(Y)}) \\
&\leq exp(-\frac{(1-p_s)^4k^2\lambda^2\ell^{*2}\delta(d)^2}{32(1-p_s)^2k\lambda(e^{-\lambda d}\ell^*+e^{-\lambda d}(1-e^{-\lambda})q+d)}) \\
Pr[X \geq E(X)-(1-p_s)^2k\lambda\ell^*\delta(d)/4] &= Pr[X \geq E(X)(1-\frac{(1-p_s)^2k\lambda\ell^*\delta(d)}{4E(X)})] \\
&\leq exp(-\frac{(1-p_s)^4k^2\lambda^2\ell^{*2}\delta(d)^2}{32E(X)+4(1-p_s)^2k\lambda\ell^*\delta(d)}) \\
&\leq exp(-\frac{(1-p_s)^4k^2\lambda^2\ell^{*2}\delta(d)^2}{32(1-p_s)^2k\lambda(e^{-\lambda d}\ell^*+e^{-\lambda d}(1-e^{-\lambda})q+d)})
\end{aligned}
$$

The last line follows from the fact that $\delta(d) \leq e^{-\lambda d}$ and $E(X) \leq (1-p_s)^2ke^{-\lambda d}(1-e^{-\lambda})^2q+\lambda d \leq (1-p_s)^2ke^{-\lambda d}(1-e^{-\lambda})\lambda q+\lambda d$. Since $e^{\lambda d}\delta(d) = 0.6(1-q+qe^{-2\lambda(1-d)}) \geq 0.6(1-q+qe^{-2\lambda})$ and any $d < d^*$, in order to ensure that both bad events have probability at most ζn^{-3}, it suffices to take

$$\frac{0.6(1-p_s)^2k\lambda(1-q+qe^{-2\lambda})\delta(d)\ell^{*2}}{32(\ell^*+(1-e^{-\lambda})q+e^{\lambda d^*}d^*)} \geq 3\log(n)+\log 1/\zeta$$

Applying the same argument to $s(a,b)-s(a,c)$ and combining both results gives $P[s(a,b)-\max(s(a,c),s(b,c)) < t] \leq 4\zeta n^{-3}$. Taking a union bound over all $\binom{n}{3} = O(n^3)$ triplets, we see that the probability of condition $i)$ failing for any triplet is at most ζ.

To get guarantees on the second condition, note that condition $i)$ implies that condition $ii)$ holds for all triplets separated by an edge of length at least ℓ^*. Thus we can focus on the triplets that are not covered

by condition i). Let $(a,b|c)$ be an arbitrary triplet such that $dist(LCA(a,b,c),LCA(a,b)) < \ell^*$ and again let $Y = s(a,b)$, $X = s(b,c)$ and d be the depth of $LCA(a,b,c)$ (note that we are focusing WLOG on $s(b,c)$ since it has the same distribution as $s(a,c)$). We want to show that with high probability, $X - Y < t$. Again, it suffices to upper bound $P[Y \leq E(Y) - t/2]$ and $P[X \geq E(X) \geq t/2]$ because $E(Y) \geq E(X)$. Note that we have already bounded the second quantity. To bound the first quantity, note that the worst case scenario is that $dist(LCA(a,b,c),LCA(a,b))$ is as small as possible, but since this quantity can be arbitrarily small, we can assume that in the worst case, $dist(LCA(a,b,c),LCA(a,b)) = 0$, which means Y has the same distribution as X. Note that this case technically cannot happen as it would imply that $\mathcal{T}$ has a trifurcating branch but it is possible to get arbitrarily close to this case with no restrictions on edge lengths. This gives:

$$\begin{aligned}
Pr[X \leq E(X) - (1-p_s)^2 k\lambda\ell^*\delta^*/4] &= Pr[X \leq E(X)(1 - \frac{(1-p_s)^2 k\lambda\ell^*\delta^*}{4E(X)})] \\
&\leq exp(-\frac{(1-p_s)^4 k^2\lambda^2\ell^{*2}\delta^{*2}}{32(1-p_s)^2 k\lambda(d + e^{-\lambda d}(1-e^{-\lambda})(1-d)^2 q)}) \\
&\leq exp(-\frac{(1-p_s)^2 k\lambda\ell^{*2}\delta^{*2}}{32(d + (1-e^{-\lambda})q)})
\end{aligned}$$

Thus, if we take:

$$k \geq \max\Big(\frac{(96\log n + 32\log 1/\zeta)(\ell^* + \lambda q + e^{\lambda d^*} d^*)}{0.6(1-p_s)^2\lambda\ell^{*2}\delta^*(1 - q + qe^{-2\lambda})}, \frac{(96\log n + 32\log 1/\zeta)(d^* + (1-e^{-\lambda})q)}{(1-p_s)^2\lambda\ell^{*2}\delta^{*2}}\Big)$$

then we have $P[X - Y \geq t] < \zeta n^{-3}$. By symmetry, this means $P[s(b,c) - s(a,b) \geq t \bigcup s(a,c) - s(a,b) \geq t] \leq 2\zeta n^{-3}$ Since we can union bound over one bad event of probability at most $2\zeta n^{-3}$ for each of the $\binom{n}{3}$ triplets, we have that conditions i) and ii) both hold with probability at least $1 - \zeta$.

A.2.2 Alternative Analysis of the Bottom-up Algorithm

Theorem 4 *The constraint on λ and q in Theorem 3 can be replaced with*

$$\lambda < \min(\frac{1 - \lambda\ell/2}{2q\gamma_2}, -\ln(1 + 2\gamma_1 + \frac{\gamma_1^2}{1-e^{-\lambda}} - \frac{1}{2q}))$$

Where $\gamma_1 = e^{-\lambda}\sqrt{c\ell} + 2\lambda^2 c\ell q_{\max}$ and $\gamma_2 = (\sqrt{\ell} + \sqrt{c} + 2\lambda c\sqrt{\ell} q_{\max})^2$. In that case, the Bottom-Up Algorithm returns the correct tree with probability at least $1 - \zeta$ if

$$k \geq \frac{e^{\lambda}(20\log n + 10\log(1/\zeta))}{\min(\lambda\ell(1 - \lambda\ell/2 - 2\lambda q\gamma_2),\ (1-e^{-\lambda})(1 - 2(1 - e^{-\lambda} + 2\gamma_1 + \frac{\gamma_1^2}{1-e^{-\lambda}})q))}$$

Note this means that as $\ell \to 0$, our constraint on λ and q becomes $\lambda q < \frac{1}{2c}$ and our bound on k approaches

$$\frac{e^{\lambda}(20\log n + 10\log(1/\zeta))}{\lambda\ell(1 - 2c\lambda q)} = O(\frac{\log n}{\ell})$$

Proof of Theorem 4: To upper bound the probabilities of the bad events, it suffices to ensure that $E(Z) > E(X)$ and bound the quantity $\frac{(E(Z)-E(X))^2}{E(Z)} = \frac{\Delta^2}{E(Z)}$. Since this quantity depends on α, we will first derive a lower bound on this quantity and determine where the minimum occurs for $\alpha \in [\ell, 1]$ as follows:

$$\begin{aligned}
\frac{\Delta^2}{E[Z]} &\geq E[Z] - 2E[X] \\
&\geq ke^{-\lambda d}(1 - e^{-\lambda\alpha} - 2(1 - e^{-\lambda(\alpha + \sqrt{c\ell})} + 2\lambda^2 c\ell q_{\max})^2 q)
\end{aligned}$$

Now let $f(\alpha) = 1 - e^{-\lambda\alpha} - 2(1 - e^{-\lambda(\alpha+\sqrt{c\ell})} + 2\lambda^2 c\ell q_{\max})^2 q)$. Next we will show that for any interval $[a, b]$, the minimum of f on $[a, b]$ occurs at either a or b. Let $y(\alpha) = e^{-\lambda\alpha}$, and let $g(y) = 1 - y - 2(1 - ye^{-\lambda\sqrt{(c\ell)}} + 2\lambda^2 c\ell q_{\max})^2 q$. Then $f = g \circ y$. Note that g' is linear with negative slope, as

$$g'(y) = -1 + 4(1 - ye^{-\lambda\sqrt{c\ell}} + 2\lambda^2 c\ell q_{\max})qe^{-\lambda\sqrt{c\ell}}$$

Since $f'(x) = -g'(e^{-\lambda x})e^{-\lambda x}$, $f'(x) = 0$ only when $g'(e^{-\lambda x}) = 0$. Since $e^{-\lambda x}$ is an increasing function, there can be at most one point where f' is 0. On the other hand, one can verify that $\lim_{x\to-\infty} f'(x) = \infty$, which means that if there is any x where $f'(x) = 0$, then f' is positive on (∞, x) and negative on (x, ∞), which means x must be a local maximum. Thus, any minimum of f on an interval $[a, b]$ can only occur on the boundaries.

$\alpha = 1$ **case:**

In the case where $\alpha = 1$, our lower bound can be written as

$$\begin{aligned} f(\alpha) &= (1 - e^{-\lambda} - 2(1 - e^{-\lambda}e^{-\lambda\sqrt{c\ell}} + 2\lambda^2 c\ell q_{\max})^2 q) \\ &\geq (1 - e^{-\lambda} - 2(1 - e^{-\lambda}(1 - \sqrt{c\ell}) + 2\lambda^2 c\ell q_{\max})^2 q) \\ &= (1 - e^{-\lambda} - 2(1 - e^{-\lambda} + e^{-\lambda}\sqrt{c\ell} + 2\lambda^2 c\ell q_{\max})^2 q) \end{aligned}$$

To make the notation easier to follow, let $\gamma_1 = e^{-\lambda}\sqrt{c\ell} + 2\lambda^2 c\ell q_{\max}$. Then we have

$$\begin{aligned} \frac{\Delta^2}{E[Z]} &\geq (1 - e^{-\lambda} - 2(1 - e^{-\lambda} + \gamma_1)^2 q) \\ &= (1 - e^{-\lambda} - 2((1 - e^{-\lambda})^2 + 2(1 - e^{-\lambda})\gamma_1 + \gamma_1^2)q) \\ &= (1 - e^{-\lambda})(1 - 2(1 - e^{-\lambda} + 2\gamma_1 + \frac{\gamma_1^2}{1 - e^{-\lambda}})q) \end{aligned}$$

Thus, in order for the bound to be non-trivial, we need $2(1 - e^{-\lambda} + 2\gamma_1 + \frac{\gamma_1^2}{1-e^{-\lambda}})q < 1$, which means

$$\begin{aligned} e^{-\lambda} &> 1 + 2\gamma_1 + \frac{\gamma_1^2}{1 - e^{-\lambda}} - \frac{1}{2q} \\ \lambda &< -\ln(1 + 2\gamma_1 + \frac{\gamma_1^2}{1 - e^{-\lambda}} - \frac{1}{2q}) \end{aligned}$$

Note that as $\ell \to 0$, the RHS approaches $\lambda < \ln(1 - \frac{1}{2q})$ which is at least $\frac{1}{2q}$, and in general, when λ satisfies the constraint above, $f(1)$ is lower bounded by a constant independent of ℓ. Also, note that the bound is bound is trival if the term inside the ln is negative.

$\alpha = \ell$ **case:**

In this case, our lower bound becomes as follows:

$$\begin{aligned} f(\alpha) &= (1 - e^{-\lambda\ell} - 2(1 - e^{-\lambda(\ell+\sqrt{c\ell})} + 2\lambda^2 c\ell q_{\max})^2 q) \\ &\geq (\lambda\ell - \frac{\lambda^2\ell^2}{2} - 2\lambda^2(\ell + \sqrt{c\ell} + 2\lambda c\ell q_{\max})^2 q) \\ &= \lambda\ell(1 - \frac{\lambda\ell}{2} - 2\lambda q(\sqrt{\ell} + \sqrt{c} + 2\lambda c\sqrt{\ell} q_{\max})^2) \end{aligned}$$

Now let $\gamma_2 = (\sqrt{\ell} + \sqrt{c} + 2\lambda c\sqrt{\ell} q_{\max})^2$. Then in order for the bound to be non-trivial, we need $\frac{\lambda\ell}{2} + 2\lambda q\gamma_2 < 1$, which means

$$\lambda < \frac{1 - \lambda\ell/2}{2q(\sqrt{\ell} + \sqrt{c} + 2\lambda c\sqrt{\ell} q_{\max})^2} = \frac{1 - \lambda\ell/2}{2q\gamma_2}$$

Note that as $\ell \to 0$, the RHS becomes $\frac{1}{2qc}$. Since $\frac{\Delta^2}{E[Z]} \geq ke^{-\lambda d} f(\alpha)$, when the constraints:

$$\lambda < \min(\frac{1-\lambda\ell/2}{2q\gamma_2}, -\ln(1+2\gamma_1+\frac{\gamma_1^2}{1-e^{-\lambda}}-\frac{1}{2q}))$$

are satisfied, and if we take

$$k \geq \frac{e^{\lambda}(20\log n + 10\log(1/\zeta))}{\min(\lambda\ell(1-\lambda\ell/2-2\lambda q\gamma_2),\ (1-e^{-\lambda})(1-2(1-e^{-\lambda}+2\gamma_1+\frac{\gamma_1^2}{1-e^{-\lambda}})q))}$$

the probability that either of the above events occur is at most $n^{-2}\zeta$. In other words, for any pair of vertices u, w that are not children of the same node, there will be a vertex u' that such that $LCA(u,u')$ is a descendent of $LCA(u,w)$ and $P[s(u,u') \leq s(u,w)] \leq 2n^{-2}\zeta$. If $s(u,u') > s(u,w)$, then (u,w) cannot be the first pair of incorrectly joined vertices. Taking a union bound over at most $n^2/2$ pairs of vertices, we see that with with probability at least $1-\zeta$, there is no first pair of incorrectly joined vertices, which means the algorithm is correct.

Since the bound on $f(\ell)$ depends on ℓ, it is general much smaller than the bound on $f(1)$, which is lower bounded by a constant. Thus, asymptotically, the bound on k is

$$\frac{e^{\lambda}(20\log n + 10\log(1/\zeta))}{\lambda\ell(1-2c\lambda q)} = O(\frac{\log n}{\ell})$$

A.3 Additional Simulations

A.3.1 Visualization of the $(\ell^*, d^*)-$Oracle:

The $(\ell^*, d^*)-$oracle presented above attempts to determine the outgroup of a triplet using the difference in the number of shared mutations between its members. In Figure S1 we visualize how well the decision rule holds for correctly and incorrectly resolved triplets. We plot $s(a,b) - \max(s(a,c), s(b,c))$ against $dist(LCA(a,b), LCA(a,b,c))$ for triplets (a,b,c) on simulated trees. The blue points show the difference between the mutations shared by the ingroup versus the mutations shared by the outgroup, and the orange points show the difference between the mutations shared by the outgroup and the ingroup.

6.6% of triplets are such that $s(a,b) - \max(s(a,c), s(b,c)) \leq t$ with (a,b) as the ingroup thus violating condition i)for $\ell^* = 0$. 0.6% of triplets are such that $s(a,b) - \max(s(a,c), s(b,c)) \leq t$ with (a,b) as the outgroup. Note that as $s(a,b) - \max(s(a,c), s(b,c)) \leq s(a,b) - s(a,c)$, requiring that $s(a,b) - \max(s(a,c), s(b,c)) < t$ is a slightly weaker condition than condition ii) and thus at most 0.6% of triplets violate it. For the set of parameters used here, t is chosen such that the probability that a triplet is indeterminable is low and the probability that the outgroup is given incorrectly is low, showing that the oracle is relatively accurate on this regime for the number of characters ($k = 50$). For for exact reconstruction of a tree though, we require that all triplets on that tree be separated by the threshold t.

As $dist(LCA(a,b), LCA(a,b,c))$ increases, this difference in the number of shared mutations between the ingroup and the non-ingroup pairs grows, making the triplets more separable. This gives us the V-shape in the figure. As the distance increases, the number of triplets that cross the threshold and thus violate either condition decreases, and after a certain point no triplets cross the threshold. This illustrates that in the result in Theorem 2 that, as we are only interested in triplets where $dist(LCA(a,b), LCA(a,b,c)) > \ell^*$ for larger values of ℓ^*, them the bound on the number of characters k needed to separate these triplets decreases.

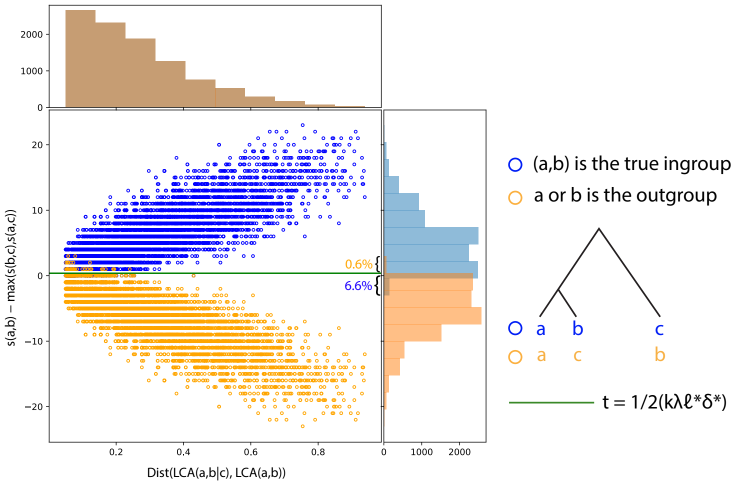

Figure S1: **Visualizing the $(\ell^*, d^*)-$Oracle Decision Rule in Simulation**. We sample 100 triplets uniformly for each of 100 trees simulated under the Asynchronous simulation framework described in Materials and Methods with $k = 50$, $\lambda = 0.5$, $q = 0.05$ and $n \approx 256$. For each triplet, we plot $s(a,b) - \max(s(a,c), s(b,c))$ for two cases: when (a,b) notates the true ingroup of the triplet (blue) and 2) when (a,b) notates one member of the ingroup and the outgroup (orange). The histograms show the density of each case for each triplet along the axes. We also report the percentage of triplets for each case that fail the decision rule (cross the threshold $t = \frac{1}{2}k\lambda\ell^*\delta^*$) for $\ell^* = 0$.

A.3.2 Validating Empirical k Found in Simulations:

In the simulations determining the empirical necessary k, we perform a coarse analysis by sampling 10 trees and determining if $\geq$ 90% of those trees satisfy the given reconstruction condition at each iteration of a binary search. To further reduce noise, we apply a logistic regression to the proportion correct for each k explored in the binary search and use the first integer k value that exceeds 90% correct according to the fitted curve. We further validate the reported empirical necessary values of k through a finer analysis. For each value of λ and q in each simulation regime and the corresponding necessary k found for those parameters, we simulated additional lineage data sets and calculated the proportion of those data sets that satisfy the reconstruction condition. We then report the difference between the proportion and the expected 0.9. The methods and results are described in Figure S2.

In Figure S2, we see that the difference in the proportions correct in the validation simulations and the expected 0.9 is relatively centered around 0, with almost all negative entries deviating from 0.9 by less than 0.1. Deviations in the negative direction are larger than deviations in the positive direction, but this is somewhat expected as there is a larger range in the distribution below 0 than above 0. In the case of the Asynchronous simulations, entries with large negative values tend to have high variance between the 10 trees, suggesting that a few outlier tree bring down the mean significantly. Overall, these results suggest that our coarse analysis yields empirical necessary values of k that are stable when expanded to a larger data set and mostly achieve or are close to achieving the intended 90% chance of meeting the reconstruction condition.

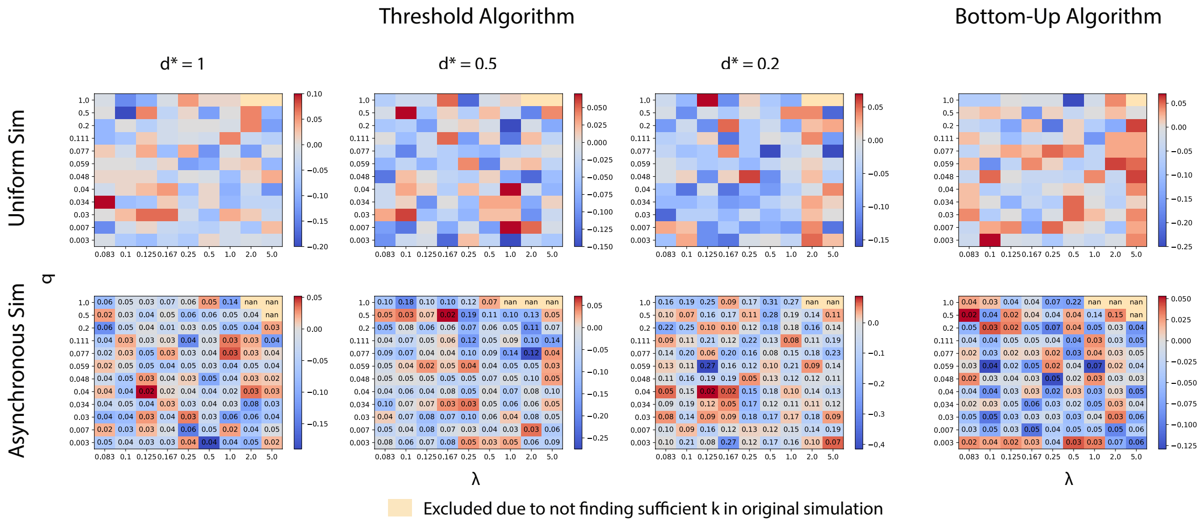

Figure S2: **Proportion of Simulated Lineage-Tracing Experiments that Meet Reconstruction Criterion for k Found in Simulation**. In the case of the Uniform simulation framework in which the topology is constant across simulated experiments, 100 lineage-tracing data sets were simulated using the given λ, q, and the corresponding empirical necessary k found for those values in simulations using the given algorithm. The difference between the proportion of data sets meeting the specified reconstruction criterion (exact or partial reconstruction at $d^* = 0.5, 0.2$) and the expected proportion of .90 is reported. In the case of the Asynchronous simulation framework, 10 trees were generated using the Asynchronous framework with 100 lineage-tracing data sets each, with each entry containing the proportion of data sets meeting the reconstruction criterion averaged across 10 trees for that λ, q and k. The number in each cell is the variance in the proportion of data sets meeting the criterion across 10 trees. The k used in each case is the k determined after applying the logistic regression.

A.3.3 Dependence on n:

To compare the asymptotic dependence of k on n in the theoretical bounds with the dependence of the necessary k in the empirical case, we simulate varying trees of size. In Figure S3 we plot the values of k in simulation against the bounds for both algorithms, in a regime where $\ell = 0.5$, $q = 0.05$. From this figure it can be seen that the bounds are tight (within a constant factor) against the empirical values and share the same shape in the dependence of k on n. These results show that the asymptotic dependence of k in the bounds for both algorithms matches the empirical dependence.

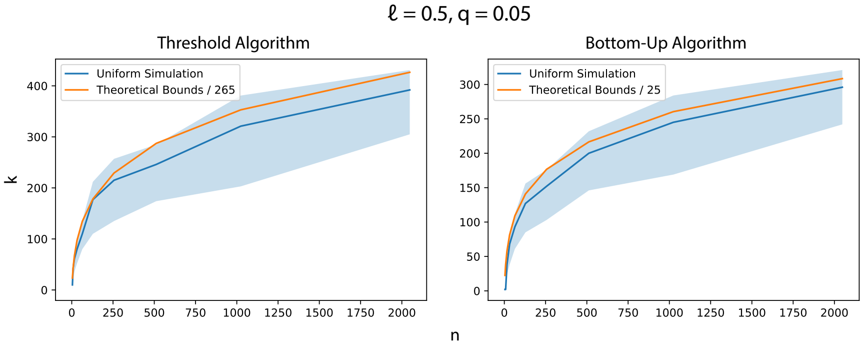

Figure S3: **Comparing the Dependence of k on n in Theory and Simulation**. The minimum necessary k given n of the Threshold Algorithm (left) and the Bottom-Up Algorithm (right) with the theoretical bounds for each case (90% of full reconstruction). Simulations performed with the uniform edge length regime for trees of size $2^2, 2^3, ..., 2^{11}$ leaves. For each value of n, edge lengths were re-scaled to be $\frac{1}{log_2(n)+1}$ to maintain uniform edge lengths. The bounds are rescaled by a constant factor, 265 for the Threshold Algorithm and 25 for the Bottom-Up Algorithm. Point-wise 95% confidence intervals are generated from the regression coefficients using the delta method, see Materials and Methods.

A.3.4 Missing Data:

Here we present simulations for the minimum k to give 0.9 probability of exact reconstruction for the case of stochastic missing data only (lemma 9). The simulations are performed with uniform edge lengths and use $p_s = 0.1$ proportion of stochastic missing data. Visualizing the bounds for lemma 9 show that indeed higher values of k are necessary to overcome the lost information (Figure S4A), consistent with the additional $(1 - p_d)^2$ term in the denominator and gap term of $e^{\lambda d^*} d^*$ when compared to the bounds of Theorem 1. Notably, the reconstruction now becomes increasingly intractable for high values of λ, due to the gap term being exponential in λ. In simulation (Figure S4B) we see that the necessary k is higher across the board, especially for values with high λ, matching the theoretical trends.

Surprisingly, unlike in lemma 2, the bound on k in lemma 9 has no asymptotic dependence on q. Taking q to be arbitrarily small (or even $q < \ell/\lambda$) causes the bound in lemma 2 to become $O(\frac{\log n}{\ell})$, yet an arbitrarily small q causes the bound in lemma 9 to remain in $O(\frac{\log n}{\ell^2})$. Examining the difference in the bounds between lemma 9 and lemma 2 (Figure S4C (Top)), we see that the difference grows larger in regions where $q < \ell/\lambda$, indicating that the bound changes asymptotically in these regions. This same pattern in the dependence on q is reflected in the empirical results (Figure S4C (Bottom)).

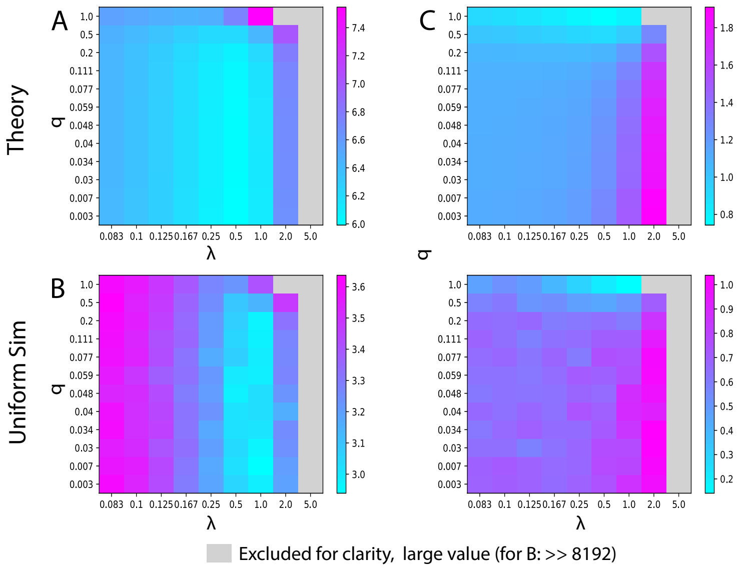

Figure S4: **Comparing the Threshold Algorithm in the Case of Stochastic Missing Data in Theory and Simulation**. The results of the Threshold Algorithm using the missing data strategy outlined in lemma 9 are shown. Simulated trees with 256 leaves, $n = 256$. Entries are log_{10} scaled. (A) Theoretical lower bound on k required for 0.9 probability of perfect tree reconstruction for varying values of q and λ in the case of missing data, with $\ell = 1/9$, $d^* = 1$, and $p_s = 0.1$. (B) Minimum k required for 0.9 probability of perfect reconstruction in simulation with a cell division topology with uniform branch lengths, $\ell = 1/9$, $d^* = 1$, and $p_s = 0.1$. (C) Scalar difference in the values of k in the case with and without missing data. Top: Difference in theoretical bounds for k for 0.9 probability of perfect reconstruction, $\ell = 1/9$, $d^* = 1$, and $p_s = 0.1$. Bottom: Difference in minimum k required for 0.9 probability of perfect reconstruction in simulation with a cell division topology with uniform branch lengths, $\ell = 1/9$, $d^* = 1$, and $p_s = 0.1$.

A.3.5 Triplets Correct:

Previous benchmarking works do not use exact reconstruction as a metric for the accuracy of phylogenetic reconstructions. A common, more relaxed metric is the triplets correct metric (or the closely related triples distance), which measures the proportion of sampled leaf triplets that are correctly (incorrectly in the case of triplets distance) inferred by the reconstructed tree [6, 40, 31]. We present the minimum k necessary in simulation for high probability of a high ($\geq 95\%$) triplets correct score on uniformly sampled triplets, showing the necessary k when exact reconstruction is not required (Figure S5). We see that the empirical necessary k decreases substantially overall compared to the case of exact reconstruction for both algorithms, showing that these algorithms can perform well in practice with a low number of characters according to traditional standards of accuracy.

Regarding the Threshold Algorithm, the reduction in the necessary k is potentially due to the fact that if triplets are sampled uniformly, most of them will have an LCA close to the root. We saw from the partial reconstruction results that the necessary k decreases across the board as d^* (the depth up to which triplets must be correct) decreases. We see also that large values of λ coincide with lower relative k in the triplets case than in the case of full reconstruction, just as in the case of $d^* << 1$. Thus, we can treat the case of uniformly sampling triplets as similar to setting a low d^*. We see that both of these effects, the lower overall necessary k and the lower k for high values of λ, also hold true of the Bottom-Up Algorithm in the case of uniformly sampling triplets. This indicates that perhaps reconstruction of triplets that diverge at the top of the tree is easier and less affected by mutation saturation in the case of the Bottom-Up Algorithm as well.

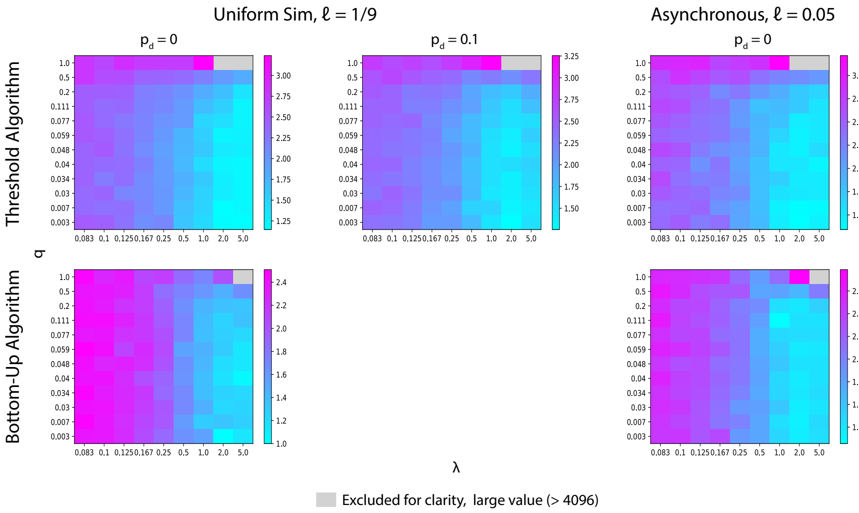

Figure S5: **Triplets Corrects Analysis for Both Algorithms**. Simulated trees with 256 leaves, $n = 256$. Entries are log_{10} scaled. Minimum k required for 0.9 probability of ≤ 0.95 proportion of 500 uniformly sampled triplets correctly reconstructed in simulation. Top row: Results for the Threshold Algorithm in the case of (From left to right) uniform edge length topology without missing data ($\ell = 1/9$, $p_s = 0.1$), uniform edge length topology with missing data ($\ell = 1/9$, $p_s = 0.1$), asynchronous topology ($\ell = 0.05$). Bottom row: Results for the Bottom-Up Algorithm in the case of (From left to right) uniform edge length topology ($\ell = 1/9$) and asynchronous topology ($\ell = 0.05$).

A.4 Additional Implementation Details:

A.4.1 Threshold Algorithm:

Due to ties in the number of shared characters, sometimes the edge-removal procedure produces more than two connected components on the sample graph G. If this occurs, we enforce a bifurcation in the tree by merging the components $C_1, C_2, ..., C_n$ into two groups. We expand the previous pseudocode as follows:

procedure SPLITSAMPLES(V)
  $G \leftarrow$ Complete graph over V
  while G is connected **do**
    $(u^*, v^*) = argmin_{(u,v)\in E}s(u, v)$
    Delete (u^*, v^*) from G
  end while
  $C_1, C_2, ..., C_n \leftarrow$ connected components of G
  if length($C_1, C_2, ..., C_n$) > 2 **then**
    $C_1, C_2 \leftarrow MergeComponents(C_1, C_2, ..., C_n)$
  end if
  $T_1, T_2 \leftarrow SplitSamples(C_1),\ SplitSamples(C_2)$
  Return binary tree with T_1 and T_2 as children of the root.
end procedure

procedure MERGECOMPONENTS($C_1, C_2, ..., C_n$)
  $LCA_1, LCA_2, ..., LCA_n \leftarrow InferParsimoniousStates(C_1, C_2, ..., C_n)$
  $m \leftarrow FindMostFrequentMutation(LCA_1, LCA_2, ..., LCA_n)$
  for i in n **do**

if m in LCA_i **then**
Add C_i to *GroupA*
else
Add C_i to *GroupB*
end if
end for
Return *GroupA*, *GroupB*
end procedure

Here, we use a naive parsimony approach. We infer the character states of the LCA of each component as the most parsimonious states given the states of the leaves in that component (a mutation appears in the LCA of a component only if it shared by all samples in that component, discounting samples with missing data at that character). Then, the mutation shared by the most LCAs is found. Heuristically, this mutation occurred early in the phylogeny and thus components sharing this mutation are more closely related. Thus, we separate components into two groups based on whether or not the LCA has this mutation.

This algorithm is implemented as the “PercolationSolver” class in the “solver” module of the Cassiopeia codebase. Here, the default arguments are used, with “joining_solver” specified as an instance of the “VanillaGreedySolver” class.

A.4.2 Bottom-Up Algorithm:

The implementation of the Bottom-Up Algorithm follows the description in the main text. If a tie occurs in the number of shared mutations between nodes, then an arbitrary pair is chosen to be merged first.

This algorithm is implemented as the “SharedMutationSolver” class in the “solver” module of the Cassiopeia codebase. Here, the default arguments are used.

A.4.3 Simulating the Asynchronous Cell-Division Topology

Note that in the trees generated by this process sister nodes need not have the same edge length and the root will have a singleton edge as in the binary case along which mutations can occur. We chose rates for the division and death waiting distributions (23.70 and 2.12, respectively) that gave an average of around 256 leaves over 1000 simulations. These rates were chosen assuming that the rate of division was 10 times that of the rate of death, and then correcting the death rate to increase the mean waiting time by $a = 0.05$ to match the shift in mean in the distribution of division times.

Due to the stochastic nature of this division process, we cannot exactly control for ℓ if we stop the experiment at a specified time. This is due to the fact that edges at the leaves of the tree may be very small if the stopping criterion is reached before the length can reach a, thus making the minimum edge length in the tree technically potentially smaller than a. We contend that these edges should not impact the analysis though. Small edges make it difficult to discern which neighboring clades are actually closer in relation. These small edges only occur at the bottom of the tree though and would only affect the edge lengths leading to single leaves, which are trivially discerned as a cherries with their neighbors, meaning that ℓ would still effectively be 0.05 in this case.

We present the pseudocode used for this simulation here:

procedure Forward Simulation($\lambda_{birth}, \lambda_{death}$)
$Lineages \leftarrow Queue()$;
$T \leftarrow$ new empty tree;
$Leaves \leftarrow \{\}$
$Lineages.push((root, None, 0))$
$SampleLineageEvent(node, parent, time, T, Leaves, Lineages, \lambda_{birth}, \lambda_{death})$
while *Lineages* is not empty **do**
$node, parent, time \leftarrow Lineages.pop()$

$SampleLineageEvent(node, parent, time, T, Leaves, Lineages, \lambda_{birth}, \lambda_{death})$
end while
Remove all nodes in T that do not have a descendant in $Leaves$
Return T
end procedure

procedure SampleLineageEvent($node, parent, time, T, Leaves, Lineages, \lambda_{birth}, \lambda_{death})$)
$t_{birth}, t_{death} \leftarrow Exp(\lambda_{birth}) + a, Exp(\lambda_{death})$;
if $time + min(t_{birth}, t_{death}) > 1$ **then**
$T \leftarrow T \cup \{(parent, node), weight = 1 - time\}$;
$Leaves \leftarrow Leaves \cup \{node\}$;
else if $t_{birth} < t_{death}$ **then**
$T \leftarrow T \cup \{(parent, node), weight = t_{birth}\}$;
$Lineages.push(Node(), node, time + t_{birth})$;
end if
end procedure

The topology simulation framework is implemented in the Cassiopeia codebase as the "BirthDeathFitnessSimulator" class in the "simulator" module. Here, "birth_waiting_distribution" is set to a lambda function that takes a rate and returns a random waiting time from an exponential distribution with that rate, shifted by 0.05. "initial_birth_scale" is set to $\approx$ 23.70. "death_waiting_distribution" is set to a lambda function that takes no arguments and returns a random waiting time from an exponential distribution with that rate $1/\approx 23.70 + 0.05$. "experiment_time" is set to 1. The other arguments are set to their defaults.

A.4.4 Simulating Single-cell CRISPR-Cas9 lineage tracing Data

The lineage tracing simulation framework is implemented in the Cassiopeia codebase as the "Cas9LineageTracingDataSimulator" class in the "simulator" module. Here, "number_of_cassettes" is set to the respective k, "size_of_cassette" is set to 1, "mutation_rate" is set to the respective λ, "state_priors" is set to a dictionary representing the q distribution, "heritable_silencing_rate" is set to 0, and "stochastic_silencing_rate" is set to 0 and 0.1, depending on whether the particular simulation has missing data. All other arguments are set to default.

A.4.5 Scoring Criterion

Full reconstruction:

We say the reconstructed tree achieves perfect reconstruction if it has a Robinson-Foulds Distance of 0, meaning the trees are isomorphic with regard to their labels.

Robinson-Foulds Distance is implemented in the codebase as the "robinson_foulds" method in the "critique" module. This method makes use of the the Ete3 package [41].

Partial Reconstruction:

To determine the sufficient k needed for exact partial reconstruction for triplets whose LCA is up to depth d, we use the same framework as in the case of full reconstruction but we change the scoring criterion. We can no longer compare ground truth and reconstructed trees by Robinson-Foulds Distance, which compares the entire tree. We instead present and show the correctness of an algorithm to determine if all triplets in a tree up to depth d are resolved correctly in the reconstructed tree. The algorithm is as follows. Let $\mathcal{T}$ be the ground truth tree, $\mathcal{T}'$ be the reconstructed tree, and d be the depth:

procedure CheckPartialReconstruction($\mathcal{T}, \mathcal{T}', d$)
CheckSplit(root($\mathcal{T}$), root($\mathcal{T}'$))
Return TRUE
end procedure

```
procedure CheckSplit(n, n', d)
    if depth(n) < d then
        return
    end if
    l, r = children(n)
    l', r' = children(n')
    if leaves_beneath(l) == leaves_beneath(l') and leaves_beneath(r) == leaves_beneath(r') then
        CheckSplit(l, l', d), CheckSplit(r, r', d)
    else
        Return FALSE
    end if
end procedure
```

Next we prove its correctness:

Claim: All triplets whose LCA is at depth $< d$ in $\mathcal{T}$ are resolved correctly in $\mathcal{T}'$ iff for every node $n < d$ in $\mathcal{T}$ there exists a node n' in $\mathcal{T}'$ whose daughter clades partition the set of leaf descendants of that node in the same way.

Proof: if: For a triplet $(a, b|c)$ whose LCA is node n, a, b must be in the clade of one daughter of n and c on the other. If there exists a node n' in $\mathcal{T}'$ that partitions the leaf nodes into the same two clades, then for each triplet whose LCA is at n, a, b will be grouped together in the same clade with c on other side, hence every triplet will be resolved correctly. If there exists n' for each $n < d$, then all triplets with LCA $< d$ will be resolved correctly. **only if:** If there is a node $n < d$ in $\mathcal{T}$ with no node n' in $\mathcal{T}'$ with an analogous partition, this implies that there is some partition of the leaves in $\mathcal{T}'$ starting from the root at or above n that does not match the partition in $\mathcal{T}$, as if all partitions were correct then n' must exist. If there is a non-matching partition, there is some partition $\{a_1, ..., a_m\}|\{b_1, ..., b_n\}$ in $\mathcal{T}$ where in $\mathcal{T}'$ there is at least one incorrect member in one of the partitioned sets: $\{a_1, ..., a'_m, b_1, ..., b'_n\}|\{a'_{m+1}, ..., a_m, b_{n'+1}, ..., b_n\}$. Then in $\mathcal{T}'$, WLOG b_1 is closer to some a_i than some b_i, and all triplets involving b_1 and a_i are incorrect in $\mathcal{T}'$. As the partition with the non-analogous partition is at depth $< d$ in $\mathcal{T}$, then some triplets whose LCA is at depth $< d$ in are incorrectly resolved in $\mathcal{T}'$.

The algorithm will find n' for every $n < d$ by matching it to a node in $\mathcal{T}'$ that has the same partition. If the algorithm does not find n' for a certain $n < d$, then it does not exist as if all partitions checked by the algorithm match up to and including n in $\mathcal{T}'$ then it must exist, and the given partition cannot be formed later as leaf descendant sets cannot add members down the tree.

Triplets Correct:

Additionally, we report the necessary k needed for 95% triplets correct in simulation. The triplets correct score is determined by sampling 1000 triplets uniformly from the ground truth tree and counting the proportion of triplets resolved accurately in the reconstructed tree.
